# Cucurbit[8]Uril Achieved Single‐Molecule Chiral Transfer to Supramolecular Circularly Polarized Luminescence

**DOI:** 10.1002/advs.75360

**Published:** 2026-04-20

**Authors:** Yongxue Li, Jie Wu, Weiheng Zhang, Hengzhi Zhang, Wei‐Lei Zhou, Yu Liu

**Affiliations:** ^1^ College of Chemistry State Key Laboratory of Elemento‐Organic Chemistry Nankai University Tianjin P. R. China; ^2^ College of Chemistry and Material Science The Technological Innovation Center of Supramolecular Chinese (Mongolian) Medicine Inner Mongolia Minzu University Tongliao P. R. China

**Keywords:** cascaded assemblies, chiral transfer, circularly polarized luminescence, cucurbit[8]uril confinement, energy transfer, multicolor luminescence, supramolecular chemistry

## Abstract

Herein, cascaded supramolecular assembly was constructed from phenylalanine‐modified biphenyl‐pyridine derivatives (*L*/*D*‐FPy), cucurbit[8]uril (CB[8]), and amphipathic sulfonatocalix[4]arene (SC4AD), which not only exhibits cascaded energy transfer and chiral transfer but also achieves amplified circularly polarized luminescence (CPL). The experimental results demonstrate that *L*/*D*‐FPy and SC4AD directly assemble through electrostatic interactions to form particles, where the luminescence intensity and quantum yield (QY) were significantly enhanced by 140 and 8 times, respectively. The positively charged *L*/*D*‐FPy and CB[8] combine to form a 1:1 stoichiometric supramolecular assembly. Compared with the *L*/*D*‐FPy@SC4AD assembly, the system with added SC4AD in the presence of CB[8] exhibited a 2.5‑fold enhancement in luminescence intensity and QY, and also amplification of chirality. Taking advantage of the energy transfer and chiral transfer, *L*/*D*‐FPy⊂CB[8]@SC4AD co‐assembly with the achiral dyes benzothiadiazole (DBT) and Nile red (NR), and the polymer polyvinyl alcohol (PVA), markedly increased the CPL. The luminescence asymmetry factor values for *L*/*D*‐FPy⊂CB[8]@SC4AD:NR were estimated to be−4.05 × 10^−3^ and+4.32 × 10^−3^ in solid film, respectively. Therefore, the present research provides an approach to construct multicolor CPL supramolecular assembly, which is not only applied to digital anti‐counterfeiting but also to chiral logic gates.

## Graphical abstract

A single‐molecule cascade supramolecular system comprising L/D‐FPy, CB[8], and SC4AD serves as an efficient light‐harvesting platform. It facilitates cascade energy transfer and chiral transfer to acceptor dyes. Co‐assembly with a PVA matrix produces a multicolor solid film that exhibits enhanced luminescence dissymmetry factors (*g*_lum_), enabling applications in digital anti‐counterfeiting and chiral logic gates.

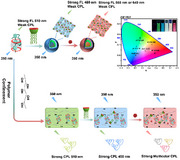

## 1 Introduction

In recent years, cascaded supramolecular assembly based on host‐guest complexes has emerged as a frontier research direction at the intersection of supramolecular chemistry and materials science, owing to its potential applications in areas such as near‐infrared cellular imaging, luminescent materials, and anti‐counterfeiting [1, 2, 3]. These applications are facilitated by energy transfer mechanisms, precise regulation of topological morphology, and amplification of chirality in supramolecular assemblies. Supramolecular chirality arises from noncovalent interactions, such as host‐guest interactions [4, 5], hydrogen bonding [6, 7], and *π*–*π* stacking [8, 9, 10], which efficiently transfer and amplify chiral information from the molecular to supramolecular levels [11, 12, 13, 14]. Taking advantage of the encapsulation of guest molecules in a macrocycle, which enables energy transfer and chiral transfer at the molecular or single‐molecule level, especially, plays a vital role in advancing the development of CPL materials for applications in chemistry [15], biology [16], and materials science [17]. The performance of CPL systems, such as those explored for 3D displays [18, 19, 20], information encryption [21, 22, 23], and biosensing [24, 25, 26], heavily depends on the absorption, emission efficiencies, as well as the luminescence asymmetry factor (*g*_lum_) of the constituent chiral substances. However, a persistent challenge lies in the typically low *g*_lum_ values of conventional CPL materials, which limit their practical applicability. In this context, macrocyclic compounds offer a promising platform for constructing advanced CPL materials by leveraging their distinctive cavity structures and superior molecular recognition capabilities.

In the construction of such functionalized supramolecular assemblies, intrinsically chiral macrocyclic host compounds such as cyclodextrins (CyDs) [27, 28, 29, 30, 31], calix[n]arenes (C[n]As) [32, 33, 34, 35, 36], pillar[n]arenes (P[n]s) [37, 38, 39, 40, 41] and others chiral macrocycles [42, 43, 44, 45, 46] serves as the preferred molecular platforms owing to their pre‐organized cavity structures, favorable biocompatibility and modifiability. For example, Professor Yang's group [30] designed and synthesized bipyrene‐substituted γ‐cyclodextrin derivatives, successfully achieving the formation of supramolecular assemblies through inclusion‐induced aggregation while significantly enhancing their chiral optical activity. Although CB[n]s lack intrinsic chromophores or chiral elements, limiting their direct use as chiral sensors, their confined cavities exhibit exceptional binding affinity and stereochemical selectivity toward guest molecules, enabling effective chirality transfer and amplification. For instance, Cao and colleagues [47] demonstrated that an achiral supramolecular organic framework (SOF) could be induced into distinct chiral conformations (M or P) via host‐guest complexation with CB[8] upon interaction with specific dipeptides (e.g., *L*‐TrpTrp or *L*‐PhePhe). The system functioned as a supramolecular chiral sensor, differentiating dipeptide sequences through characteristic circular dichroism (CD) responses. Building on this principle of CB[n]‐mediated chiral induction, our group explored a cascade assembly strategy [48]. We found that CB[8] encapsulation of cationic paddle‐like phenothiazine derivatives not only facilitated the formation of supramolecular frameworks with enhanced near‐infrared (NIR) fluorescence but also enabled subsequent chirality transfer upon co‐assembly with *L*/*D*‐tripeptides. This process yielded mirror‐image CD signals and further amplified NIR emission, ultimately achieving thermally responsive and reversible chiral assemblies. We also discovered that energy transfer in single molecules confined by macrocycles can achieve controllable topological morphology transformation, which has been successfully applied to targeted imaging of cancer cell mitochondria [49]. While numerous reports exist on energy transfer and chiral transfer confined by macrocycles, however, the enhancement of CPL through the chiral transfer of single molecules confined by macrocycles to supramolecular assemblies has rarely been reported, to the best of our knowledge.

**SCHEME 1:**
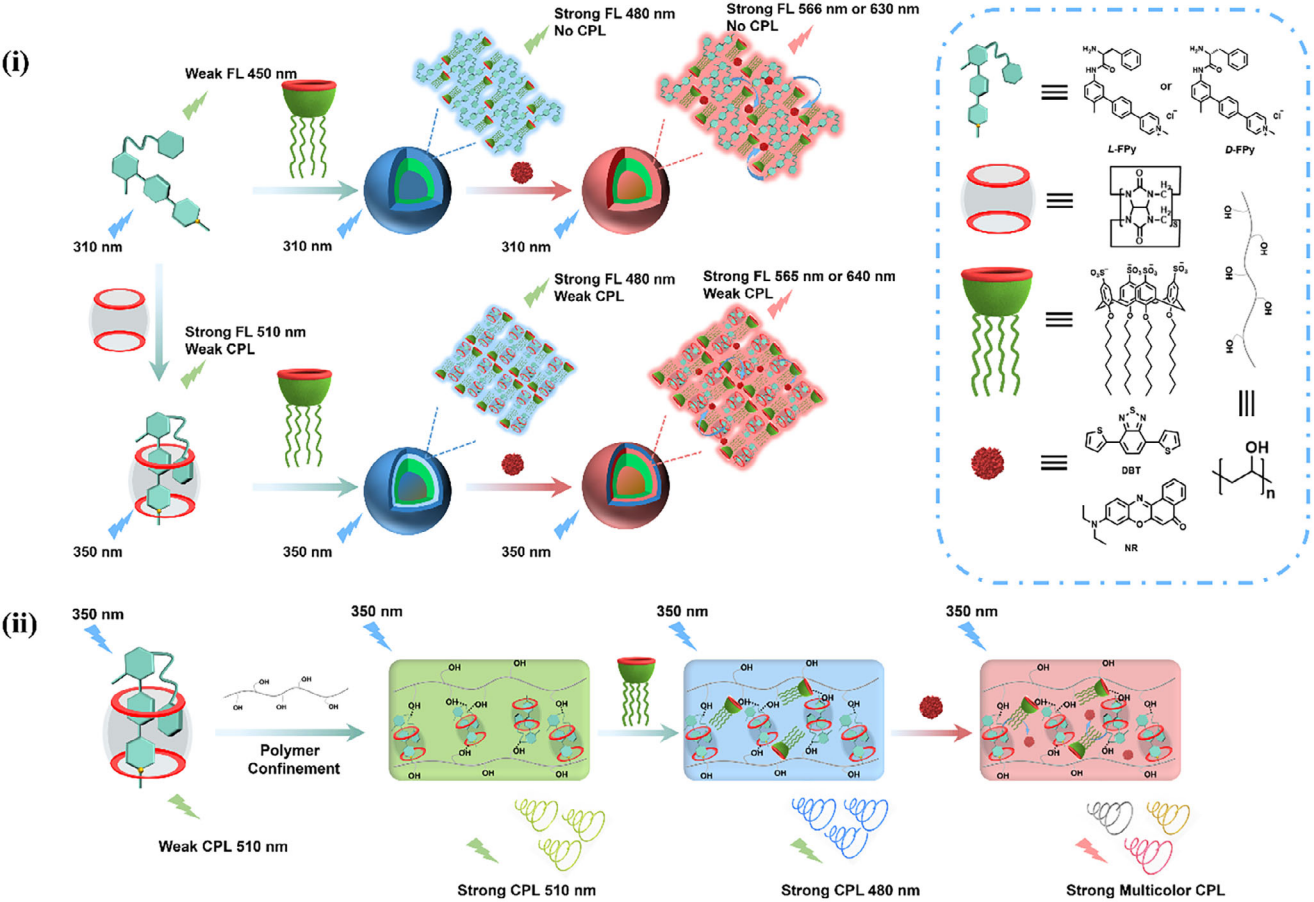
Schematic illustration of the process of single‐molecule chiral transfer to the supramolecular enhancement of CPL assemblies (i) in solution (ii) in solid films.

Herein, we constructed a single‐molecule cascaded fluorescence resonance energy transfer and chiral transfer system via a secondary assembly strategy using two types of macrocyclic molecules, CB[8] and amphipathic p‐sulfonatocalix[4]arene octyl ether (SC4AD) (Scheme 1). Phenylalanine‐modified biphenyl‐pyridine derivatives (*L*/*D*‐FPy) were encapsulated within the cavity of CB[8], and an amplified and transferred CD signal was observed in the inclusion complex *L*/*D*‐FPy⊂CB[8] due to the formation of a self‐assembly through charge‐transfer (CT) interactions within the confined cavity of CB[8] [50, 51]. Simultaneously, the nearly invisible fluorescence emission of *L*/*D*‐FPy was greatly enhanced by approximately 90‐fold upon encapsulation by CB[8], exhibiting green fluorescence and CPL (*g*_lum_ = ± ∼10^−4^) at 510 nm. Furthermore, the *L*/*D*‐FPy⊂CB[8] complex was further assembled with SC4AD to form spherical nanoparticles with a diameter of approximately 200 nanometers. The fluorescence emission intensity and QY of these nanoparticles were four times higher than those of *L*/*D*‐FPy⊂CB[8]. This phenomenon is primarily attributed to the close‐packed structure formed by co‐assembly with amphipathic SC4AD, which immobilized the phosphors to restrict nonradiative relaxation pathways and shielded them from quenchers to some extent. The resulting supramolecular assemblies (*L*/*D*‐FPy⊂CB[8]@SC4AD) served as an excellent light‐harvesting platform, transferring energy to benzothiadiazole (DBT) and Nile red (NR) dyes with energy transfer efficiencies of 40.7% and 37.1%, and 58.8% and 59.0%, respectively. To further enhance the functionality of the assembly, *L*/*D*‐FPy⊂CB[8]@SC4AD was co‐assembled with the polymer polyvinyl alcohol (PVA) to form a solid film, which markedly enhanced the CPL and the *g*_lum_. The *g*_lum_ values for *L*/*D*‐FPy⊂CB[8]@SC4AD were estimated to be −1.7 × 10^−3^ and + 1.07 × 10^−3^, respectively. Taking advantage of the energy transfer and chiral transfer from the *L*/*D*‐FPy⊂CB[8]@SC4AD assembly to the achiral dyes DBT and NR, multicolor luminescence and multicolor CPL were successfully achieved, and applied to digital anti‐counterfeiting and chiral logic gates.

## 2 Results and Discussion

A pair of phenylalanine‐modified biphenyl‐pyridine derivatives was successfully synthesized from 6‐methyl‐4'‐(pyridin‐4‐yl)‐[1,1'‐biphenyl]‐3‐amine and Boc‐protected phenylalanine isomers via an amide condensation reaction (Scheme S1). The structures of the guest molecules (*L*/*D*‐FPy) were characterized by nuclear magnetic resonance (^1^H NMR, ^1^ ^3^C NMR) and high‐resolution mass spectrometry (Figures S1–S19). After synthesizing the guest molecules *L*/*D*‐FPy, the host‐guest recognition behavior with CB[8] was first investigated. ^1^H NMR titration (Figures S20 and S21) and 2D ^1^H‐^1^H COSY experiments (Figures S22 and S23) indicated that the aromatic group on phenylalanine and some protons on the biphenyl‐pyridinium unit (Ha‐c) in *L/D‐*FPy exhibited upfield chemical shifts due to host‐guest shielding effects, indicating that the aryl group on phenylalanine and part of the biphenyl‐pyridinium (Ha‐c) unit are enclosed within the hydrophobic cavity of CB[8]. However, the phenyl protons (Hd‐g) reside in the deshielding region of CB[8], resulting in a downfield shift. Therefore, in *L*/*D‐*FPy, the positively charged biphenyl‐pyridinium group and the aromatic unit on phenylalanine form a face‐to‐face π‐π stacking structure via deep encapsulation within the macrocycle cavity [52]. Meanwhile, compared to CB[8], CB[7] possesses a smaller hydrophobic cavity capable of binding only a single guest molecule [53]. ^1^H NMR titration (Figures S24 and S25) indicated the aromatic group on phenylalanine and all protons on the biphenyl‐pyridinium unit of *L*/*D‐*FPy exhibited upfield shifts in the presence of CB[7], indicating that CB[7] encapsulates both the aryl group of phenylalanine and the biphenyl‐pyridinium (Ha‐d) moiety of *L*/*D*‐FPy, respectively. Furthermore, 2D NOESY NMR experiments confirmed that the phenylalanine and Ha‐d of *L*/*D*‐FPy were encapsulated by CB[7] to form host‐guest (Figures S26 and S27).

Additionally, the 2D ^1^H‐^1^H COSY and NOESY NMR spectra of *L*/*D*‐FPy⊂CB[8] revealed that the aryl group on phenylalanine and the partial biphenyl‐pyridinium unit (Ha‐c) are enclosed within the cavity of CB[8] (Figures S28–S31). This confirmed that the encapsulation mode of *L*/*D*‐FPy⊂CB[8] corresponds to an inclusion complex.

**FIGURE 1:**
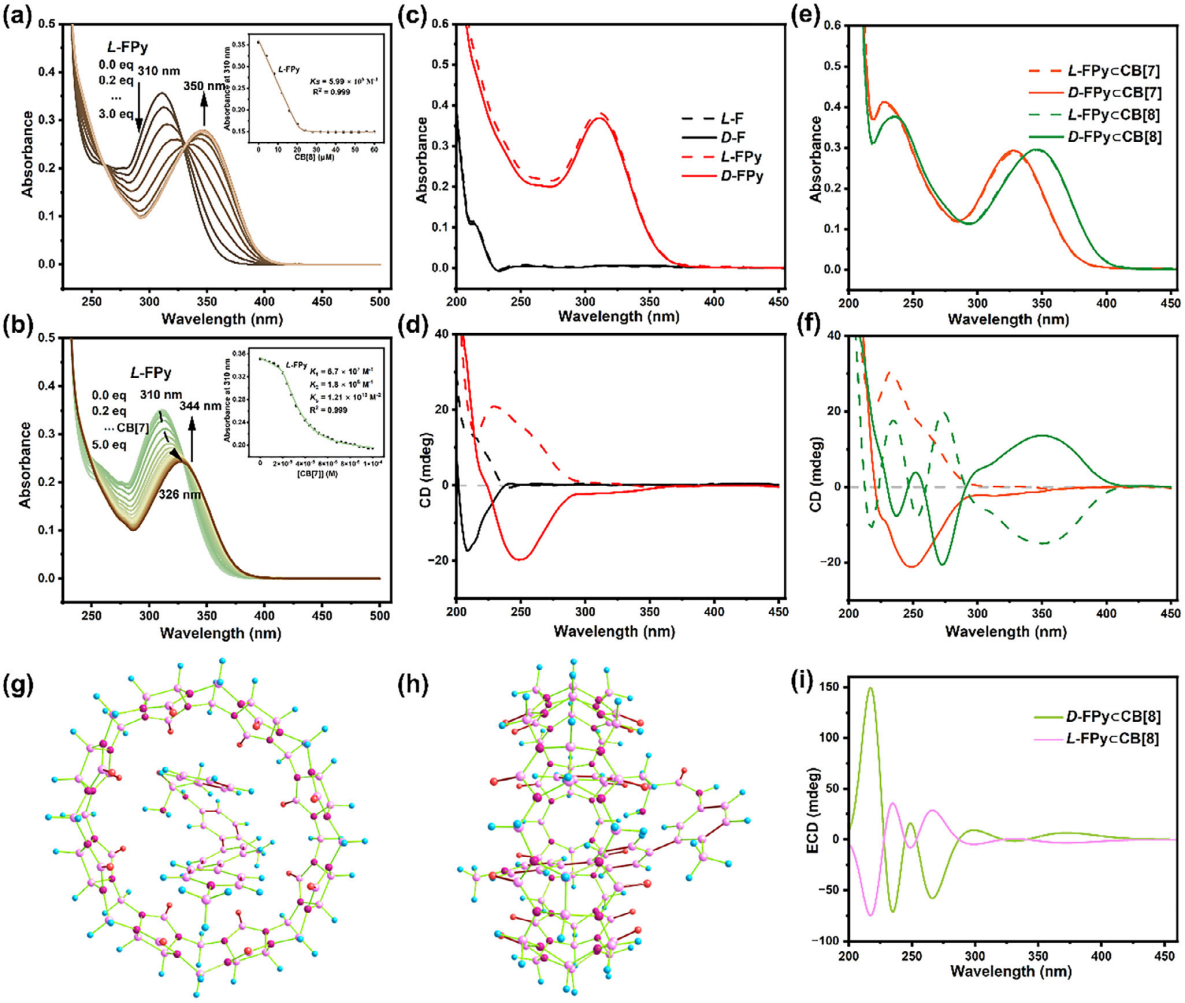
(a) UV–vis absorption spectra and absorbance intensity changes of *L*‐FPy at 310 nm (inset) upon addition of CB[8] in H_2_O at 298 K. (b) UV–vis absorption spectra and absorbance intensity changes of *L*‐FPy at 310 nm (inset) upon addition of CB[7] in H_2_O at 298 K. (*L*/*D*‐FPy] = 2.0 × 10^−5^ m, [CB[7]] = 0−1.0 × 10^−4^ m and [CB[8]] = 0−6.0 × 10^−5^ m). (c) UV–vis and (d) CD spectra of *L*/*D*‐F (*L*/*D*‐phenylalanine), (e) UV–vis and (f) CD spectra of *L*/*D*‐FPy⊂CB[7] and *L*/*D*‐FPy⊂CB[8]. ([*L*/*D*‐F] = *L*/*D*‐FPy] = 2.0 × 10^−5^ m, [CB[7]] = 4.0 × 10^−5^ m and [CB[8]] = 2.0 × 10^−5^ m). (g,h) Optimized structure of *L*‐FPy⊂CB[8]. (i) Stimulated CD spectrum based on optimized structure of *L*/*D*‐FPy⊂CB[8].

UV–vis spectroscopy experiments were also performed to determine the binding constants and stoichiometric ratios. Job's plot analysis revealed an inflection point at a molar fraction of 0.5 (Figure S34), indicating a 1:1 host‐guest stoichiometry for the complex between *L*/*D*‐FPy and CB[8]. The stoichiometry was further confirmed by high‐resolution mass spectrometry (HR‐MS) (Figures S32 and S33). For the *L*‐FPy⊂CB[8] complex, a peak at m/z 875.8100 was assigned to the [M−Cl^¯^+H^+^]^2+^/2 species (calcd. **875**.8113). A corresponding peak at the same m/z value was observed for the *D*‐FPy⊂CB[8] complex. These results confirmed the formation of a 1:1 inclusion complex between *L*/*D*‐FPy and CB[8]. The host‐guest binding behavior between *L*/*D*‐FPy and CB[8] was monitored by UV–vis titration. As shown in Figure 1a and Figure S36a, the characteristic absorption band of free *L*/*D*‐FPy at 310 nm exhibited a bathochromic shift and a decrease in intensity upon the stepwise addition of CB[8]. Two clear isosbestic points appeared at 262 and 330 nm. These spectral changes suggested the formation of a face‐to‐face *π*–*π* stacking structure between the benzene moiety of phenylalanine and the biphenyl‐pyridine unit within the CB[8] cavity, which could facilitate a host‐stabilized intramolecular charge‐transfer (ICT) interaction. The association binding constants (Ka) for the 1:1 *L*/*D*‐FPy⊂CB[8] complexes in aqueous solution were determined by nonlinear least‐squares fitting of the UV–vis titration data, yielding values of 5.99 × 10^6^ m ^−^^1^ and 9.02 × 10^6^ m ^−^^1^, respectively (insets in Figure 1a; Figure S36a). In contrast, CB[7] possesses a smaller hydrophobic cavity capable of binding only a single guest molecule per host [34, 36, 37]. The interaction between *L*/*D*‐FPy and CB[7] was also studied by UV–vis spectroscopy. The Job's plot for this system showed an inflection point at a molar fraction of 0.33 (Figure S35), corresponding to a 1:2 (host: guest) binding stoichiometry. The changes in the UV–vis spectra at 310 nm (Figure 1b; Figure S36b) were analyzed. The binding constant (Ks) for the 1:2 complexes of CB[7] with *L*‐FPy and *D*‐FPy were calculated by nonlinear least‐squares fitting as 1.21 × 10^1^ ^2^ m ^−^^2^ and 6.5 × 10^1^ ^2^ m ^−^^2^, respectively (Figure 1b; Figure S36b, inset).

Circular dichroism spectroscopy, a technique based on the differential absorption of left‐ and right‐handed circularly polarized light by chiral substances, was employed to probe the chiroptical properties of the guest molecules *L*/*D*‐FPy and their host‐guest complexes. As shown in Figure 1c,d, free *L*‐FPy and *D*‐FPy exhibited mirror‐symmetrical CD signals with a crossover around 214 nm. Upon conjugation of the chiral phenylalanine moiety to the biphenyl‐pyridine luminophore in *L*/*D*‐FPy, a distinct absorption band emerged at 310 nm, accompanied by corresponding CD activity. Specifically, *L*‐FPy showed positive Cotton effects at approximately 250 and 310 nm, whereas its enantiomer *D*‐FPy displayed negative signals at the same wavelengths, suggesting the presence of π→π^*^ or n→π^*^ electronic transitions associated with these bands. Notably, the host‐guest complex *L*‐FPy⊂CB[8] exhibited four Cotton effects at 234, 253, 274, and 350 nm, with *D*‐FPy⊂CB[8] giving a mirror‐image CD spectrum (Figure 1e,f). This significant amplification and modulation of the CD signal is attributed to the well‐defined supramolecular geometry within the CB[8] cavity. As previously outlined, the electron‐deficient biphenyl‐pyridinium moiety and the electron‐rich aryl unit of phenylalanine in *L*/*D*‐FPy engage in a face‐to‐face *π*–*π* stacking interaction facilitated by charge transfer, forming within the CB[8] cavity. The spatial confinement of the macrocycle effectively transfers and amplifies the inherent chirality from the amino acid to the entire CT complex. In contrast, this pronounced chiroptical enhancement was not observed for complexes with CB[7], consistent with its different, non‐cooperative binding mode that precludes the formation of an analogous stacked structure. Consequently, further detailed chiroptical studies with CB[7] were not conducted. These results demonstrate that the macrocyclic host cavity not only encapsulates the chiral guest but also effectively modulates, amplifies, and transfers its chiroptical properties, providing crucial spectroscopic evidence and a design principle for constructing advanced materials with circularly polarized luminescence functionality. Theoretical calculations were also carried out for the complex *L*/*D*‐FPy⊂CB[8]. Based on the optimized structure of *L*/*D*‐FPy⊂CB[8], the simulated CD spectrum is in agreement with the experimental result (Figure 1g–i; Figure S37).

**FIGURE 2:**
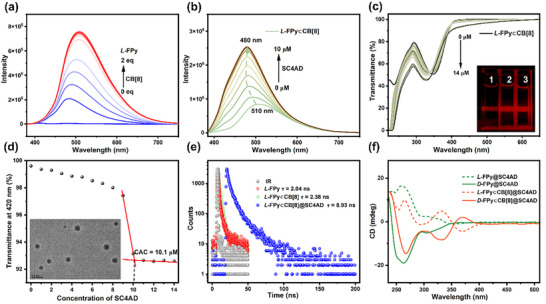
(a) Fluorescence emission spectral changes of *L*‐FPy upon addition of 0, 0.2, 0.4, 0.6, 0.8, 1.0, 1.2, 1.4, 1.6, 1.8, and 2.0 equivalent CB[8] in H_2_O at 298 K. ([*L*‐FPy] = 2.0 × 10^−5^ m, 𝜆_ex_ = 350 nm). (b) Fluorescence emission spectrum of *L*‐FPy⊂CB[8] ([*L*‐FPy] = [CB[8]] = 2.0 × 10^−5^ m) upon the addition of 0−10 × 10^−6^ m SC4AD in aqueous solution. (c) Transmittance changes of *L*‐FPy⊂CB[8] in varying concentration of SC4AD from 0 to 14 × 10^−6^ m. Inset: Tyndall effect of *L*‐FPy (1), *L*‐FPy⊂CB[8] (2), and *L*‐FPy⊂CB[8]@SC4AD (3). (d) Dependence of the optical transmittance at 420 nm upon the addition of SC4AD. Inset: Transmission electron microscopy image of *D*‐FPy⊂CB[8]@SC4AD assembly. (e) Time‐resolved photoluminescence decay curves of *D*‐FPy, *D*‐FPy⊂CB[8] and *D*‐FPy⊂CB[8]@SC4AD in aqueous solution at 298 K. (f) CD spectra of *L*/*D*‐FPy@SC4AD and *L*/*D*‐FPy⊂CB[8]@SC4AD. (*L*/*D*‐FPy] = 1.0 × 10^−4^ m, [CB[8]] = 1.0 × 10^−4^ m and [SC4AD] = 5.0 × 10^−5^ m).

Following the confirmation of the binding mode between *L*/*D*‐FPy and CB[8] through the aforementioned experiments, we further investigated the optical properties of the corresponding host‐guest complex using photoluminescence spectroscopy in aqueous solution. Specifically, a weak fluorescence emission peak was observed at 450 nm for *L*‐FPy alone. Upon continuous addition of CB[8] to the *L*‐FPy solution, a distinct new fluorescence emission peak appeared at 510 nm (Figure 2a). A similar phenomenon was observed when the CB[8] solution was gradually added to the *D*‐FPy solution (Figure S38a). It is reported that sulfonic acid calix[4]arenes with inherent amphiphilicity can be used to construct multivalent supramolecular assembly systems, thereby significantly enhancing the fluorescence and phosphorescence emission intensification of organic luminescent molecules in the aqueous phase. After elucidating the fluorescence emission behavior of the complex formed between the guest molecule and CB[8], we aimed to enhance the luminescence by introducing amphiphilic sulfonated calix[4]arene to promote its secondary assembly with *L*/*D*‐FPy⊂CB[8]. As shown in Figure 2b, upon adding SC4AD, the fluorescence intensity at 510 nm progressively blue‐shifted to 480 nm, indicating effective co‐assembly of SC4AD with *L*‐FPy⊂CB[8] and a further four‐fold increase in fluorescence intensity. Analogous fluorescent changes were also observed in the complex *D*‐FPy⊂CB[8] (Figure S38b). To investigate the mechanism behind this fluorescence enhancement, we compared the fluorescence behavior of the guest alone with that of SC4AD. In the absence of CB[8], SC4AD still enhances *L*/*D*‐FPy fluorescence by over 140‐fold, and continued addition of SC4AD does not cause fluorescence quenching (Figure S39). This indicates that electrostatic assembly between *L*/*D*‐FPy and SC4AD was the primary mechanism for fluorescence enhancement. Furthermore, the assembly process between SC4AD and *L*/*D*‐FPy⊂CB[8] as well as *L*/*D*‐FPy was investigated by monitoring changes in optical transmittance at 420 and 380 nm wavelengths, respectively. Results show that when SC4AD was gradually added to the solution containing *L*‐FPy⊂CB[8] (or *D*‐FPy⊂CB[8]) complexes, optical transmittance initially exhibits only minor changes before gradually decreasing. The critical aggregation concentration of SC4AD was 10 µm, indicating the formation of ternary nanoclusters via macrocyclic confinement and electrostatic interactions in solution (Figure 2c,d; Figure S40). In the absence of CB[8], the optical transmittance of *L*‐FPy (or D‐FPy) gradually decreased with increasing SC4AD concentration. Similarly, when the SC4AD concentration reached 9.3 µm, no significant changes in transmittance were observed beyond the critical aggregation concentration (Figure S41). The optical transmittance measured at 380 nm versus *L*‐FPy (or *D*‐FPy) concentration curve indicates that SC4AD effectively induces *L*/*D*‐FPy aggregation. Through *π*–*π* stacking interactions and electrostatic interactions, it forms a stable assembly system, significantly reducing the critical aggregation concentration (CAC). Based on CAC calculations, the optimal mixing ratio of SC4AD with *L*/*D*‐FPy⊂CB[8] and *L*/*D*‐FPy was approximately 1:2, yielding the lowest transmittance and pronounced Tyndall effect (Figure 2c; Figures S40a and S41a,c, inset). Continued addition of SC4AD no longer enhances transmittance, indicating that the optimal mixing ratio remains constant and the co‐assembly system exhibits high stability. The fluorescence intensity experiments of *L*/*D*‐FPy⊂CB[8]@SC4AD assembly at room temperature within 24 h have been performed to further indicate the stability of the assembly (Figure S42).

Subsequently, the morphological structure after assembly was analyzed by transmission electron microscopy (TEM). The results revealed that *L*/*D*‐FPy@SC4AD self‐assembled into numerous irregular aggregates with diameters of approximately 40 nm (Figure S41b,d, inset), whereas *L*/*D*‐FPy⊂CB[8]@SC4AD existed as densely packed spherical nanoparticles with a particle size of about 200 nm (Figure 2d; Figure S40b, inset). Concurrently, the fluorescence QY of *L*‐FPy and *L*‐FPy⊂CB[8] were measured to be 0.53% and 2.92%, respectively (*D*‐FPy: 0.83%, *D*‐FPy⊂CB[8]: 2.93%) (Figure S43). In contrast, the QY of *L*/*D*‐FPy@SC4AD and *L*/*D*‐FPy@CB[8]@SC4AD increased significantly to 4.35%/4.48% and 11.49%/11.83%, respectively (Figure S44). This enhancement originated from the strong binding affinity between CB[8] and *L*/*D*‐FPy. Specifically, *L*/*D*‑FPy first entered the cavity of CB[8] via host‐guest interactions, followed by assembly with SC4AD through electrostatic interactions to form nanoparticles. Under this macrocyclic confinement and secondary assembly, the QY was markedly improved. In contrast, *L*‐FPy and *D*‐FPy form smaller nanoparticles with SC4AD solely through electrostatic interactions, resulting in relatively lower QYs. The fluorescence lifetimes of *L*/*D*‐FPy, *L*/*D*‐FPy@SC4AD, *L*/*D*‐FPy⊂CB[8], and *L*/*D*‐FPy⊂CB[8]@SC4AD were measured to be 2.04 (1.89), 8.57 (8.39), 2.38 (2.39), and 8.93 (8.37) ns, respectively (Figure 2e; Figure S45). To investigate the assembly mechanism, we further tested the circular dichroism spectra of the assemblies. As shown in Figure 2f, the assembly formed between SC4AD and *L*/*D*‐FPy via electrostatic interactions exhibits no discernible change in circular dichroism signal in the absence of CB[8], indicating that *L*/*D*‐FPy was not encapsulated within the SC4AD cavity. In contrast, the assembly *L*/*D*‐FPy⊂CB[8]@SC4AD exhibits mirror‐symmetric circular dichroism signals at 250, 310, and 350 nm, demonstrating that the assembly driving force primarily relies on host‐guest inclusion by CB[8] together with electrostatic interactions.

**FIGURE 3:**
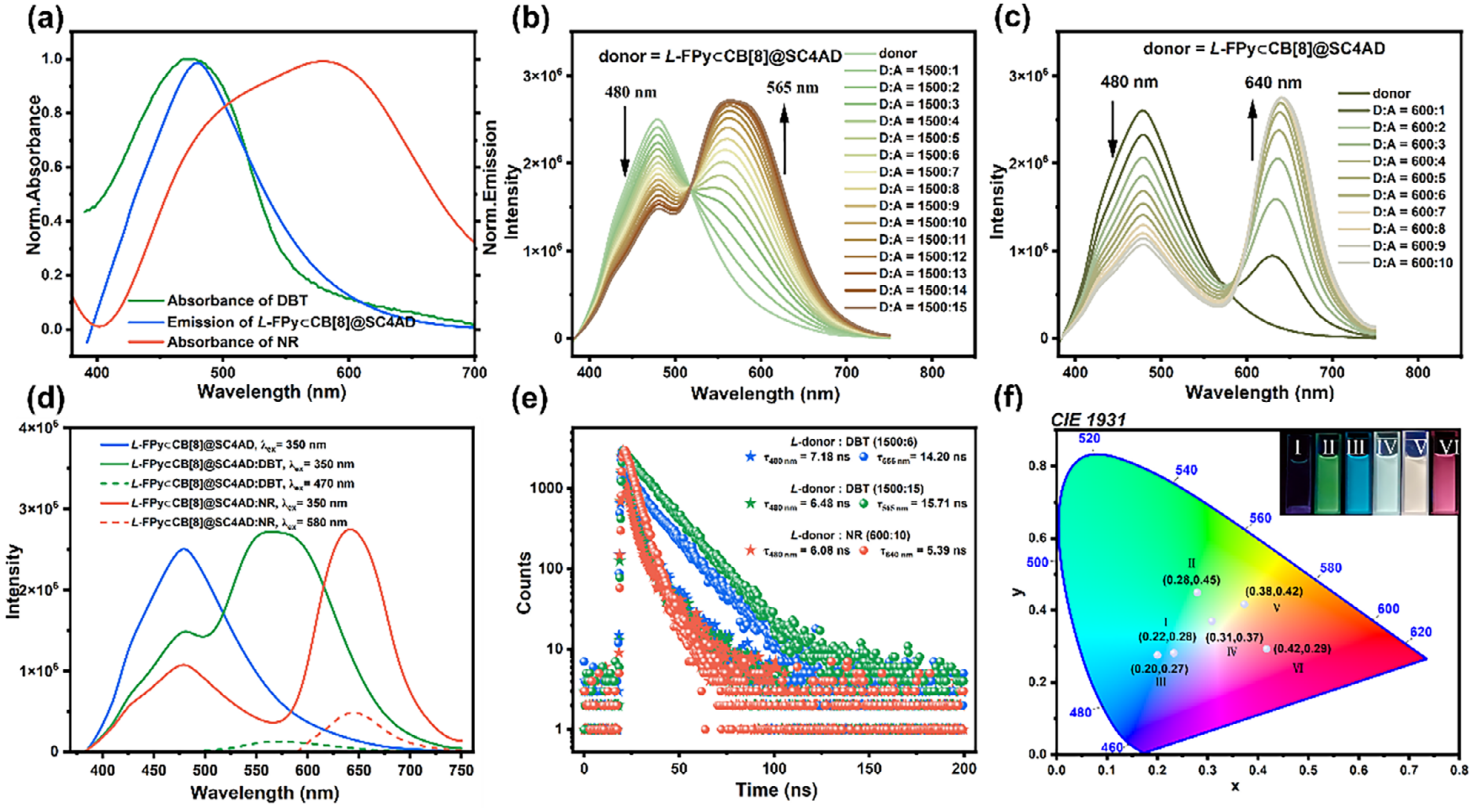
(a) Normalized emission spectrum of *L*‐FPy⊂CB[8]@SC4AD assembly and absorption spectra of DBT, NR. (b) Fluorescence emission spectrum of *L*‐FPy⊂CB[8]@SC4AD:DBT at different donor/acceptor ratios in aqueous solution at 298 K. (c) Fluorescence emission spectrum of *L*‐FPy⊂CB[8]@SC4AD:NR at different donor/acceptor ratios in aqueous solution at 298 K. (d) Fluorescence emission spectra of *L*‐FPy⊂CB[8]@SC4AD (λ_ex_ = 350 nm), *L*‐FPy⊂CB[8]@SC4AD:DBT (λ_ex_ = 350 nm), *L*‐FPy⊂CB[8]@SC4AD:DBT (λ_ex_ = 470 nm), *L*‐FPy⊂CB[8]@SC4AD:NR (λ_ex_ = 350 nm) and *L*‐FPy⊂CB[8]@SC4AD:NR (λ_ex_ = 580 nm) in aqueous solution at 298 K. (e) Time‐resolved photoluminescence decay curves of *L*‐FPy⊂CB[8]@SC4AD:DBT and *L*‐FPy⊂CB[8]@SC4AD:NR in aqueous solution at 298 K. (f) CIE 1931 chromaticity diagram and inset: Photographs of the aqueous solutions of *L*‐FPy (I), *L*‐FPy⊂CB[8] (II), *L*‐FPy⊂CB[8]@SC4AD (III), *L*‐FPy⊂CB[8]@SC4AD:DBT (IV, V), *L‐*FPy⊂CB[8]@SC4AD:NR (VI). ([*L*‐FPy] = [CB[8]] = 2.0 × 10^−5^ m, [SC4AD] = 10 × 10^−6^ m, [DBT] = 2.0 × 10^−7^ m, [NR] = 3.33 × 10^−7^ m, λ_ex_ = 350 nm).

Given the exceptional luminescent properties of this assembly, we anticipated that it would serve as an ideal donor for constructing light‐harvesting systems in aqueous solutions. We selected two highly fluorescent dyes, DBT and NR, as acceptors for the following reasons: First, their absorption bands significantly overlapped with the emission spectrum of the *L*/*D*‐FPy⊂CB[8]@SC4AD assembly (Figure 3a; Figure S46a). Second, these dyes could be readily encapsulated within the close‐packed structure of the *L*/*D*‐FPy⊂CB[8]@SC4AD assembly via hydrophobic interactions, meeting the requirement for short‐distance fluorescence resonance energy transfer (FRET) between the donor and acceptor. As shown in Figure 3b and Figure S46b, upon the stepwise addition of DBT, the fluorescence intensity of *L*/*D*‐FPy⊂CB[8]@SC4AD at 480 nm gradually decreased, while the emission intensity at 565 nm progressively increased. White‐light‐like emission was observed at a donor‐to‐acceptor ratio of 1500:6, and when the ratio reached 1500:15, the emission intensities of both donor and acceptor remained nearly constant. Energy transfer efficiency (Φ_ET_) and antenna efficiency (AE) are two key metrics for evaluating the performance of this light‐harvesting system. At a donor‐to‐acceptor ratio of 1500:15, the measured Φ_ET_ values for *L*/*D*‐FPy⊂CB[8]@SC4AD:DBT were 40.7% and 37.1% (Figure 3d; Figure S46c). At the same ratio, the calculated AE values were 18.2 and 17.9 (Figure S47). In contrast, the emission spectrum of the *L*/*D*‐FPy@SC4AD assembly exhibited significant overlap with the absorption spectra of DBT and NR (Figure S48a). The *L*/*D*‐FPy@SC4AD assembly also served as a platform for light harvesting and energy transfer to DBT (Figure S48b,c), with measured Φ_ET_ of 55.7% and 54.3% and AE values of 17.8 and 23.1 (Figures S48d and S49). Further evidence for efficient FRET was provided by changes in the time‐resolved fluorescence decay curves before and after energy transfer. As shown in Figure 3e and Figure S50a,b, when the donor‐to‐acceptor molar ratio reached 1500:15, the fluorescence lifetime of the *L*/*D*‐FPy⊂CB[8]@SC4AD:DBT system at 480 nm shortened to 6.48 and 6.54 ns. Under nearly identical donor‐to‐acceptor ratios, the fluorescence lifetime of the *L*/*D*‐FPy@SC4AD:DBT system at 480 nm shortened to 5.13 and 5.02 ns (Figure S50c). The CIE 1931 chromaticity diagram and photographs of aqueous solutions were shown in Figure 3f and Figure S46d. The fluorescence QY of *L*/*D*‐FPy⊂CB[8]@SC4AD:DBT and *L*/*D*‐FPy@SC4AD:DBT was measured at donor:acceptor = 1500:15 as 26.00% (23.19%) and 16.76% (16.29%), respectively (Figures S51a,b and S52a,b). The fluorescence QY for *L*/*D*‐FPy⊂CB[8]@SC4AD:DBT at a donor:acceptor ratio of 1500:6 was determined to be 17.56% and 17.55%, respectively (Figure S51c,d). The above results are consistent with a FRET mechanism from the donor to the acceptor.

Furthermore, to demonstrate the generality of the *L*/*D*‐FPy⊂CB[8]@SC4AD assembly as a light‐harvesting platform, we replaced the acceptor DBT with NR (Figure 3a; Figure S46a). As shown in Figure 3c and Figure S54, upon the gradual addition of NR to the *L*/*D*‐FPy⊂CB[8]@SC4AD assembly solution, the donor fluorescence intensity at 480 nm decreased, while the emission intensity at 640 nm increased significantly. A similar trend was observed for the *L*/*D*‐FPy@SC4AD:NR assemblies (Figure S55). At a donor‐to‐acceptor molar ratio of 600:10, the fluorescence lifetime of the *L*/*D*‐FPy⊂CB[8]@SC4AD:NR system at 480 nm shortened to 6.08 and 5.82 ns (Figure 3e; Figure S50a). According to the fluorescence quenching of the *L*/*D*‐FPy⊂CB[8]@SC4AD during the energy transfer process, the measured Φ_ET_ values at a donor‐to‐acceptor ratio of 600:10 was 58.8% and 59.0% (Figure 3d; Figure S46c), with AE values of 5.6 and 5.6 (Figure S53).

Similarly, for the *L*/*D*‐FPy@SC4AD:NR assembly at a ratio of 600:10, the Φ_ET_ values were 64.5% and 65.9% (Figure S56), with AE values of 7.0 and 7.9 (Figure S57). The fluorescence lifetime of this system at 480 nm shortened to 4.32 and 4.31 ns (Figure S50d). The fluorescence QY of *L*/*D*‐FPy⊂CB[8]@SC4AD:NR and *L*/*D*‐FPy@SC4AD:NR were measured as 36.33% (35.90%) and 14.02% (15.05%), respectively (Figures S51e,f and S52c,d). In control experiments, neither the *L*/*D*‐FPy⊂CB[8]:DBT nor the *L*/*D*‐FPy⊂CB[8]:NR systems showed emission peaks at 565 or 640 nm. This indicates that energy transfer between *L*/*D*‐FPy⊂CB[8] and the fluorescent dyes (DBT or NR) required multivalent assembly with SC4AD (Figure S58), laying the groundwork for subsequent energy transfer from chiral donors to achiral acceptors within the assembly. Additionally, the CD spectra of the assemblies in the donor‐acceptor systems were recorded (Figure S59). At the experimental concentrations, the UV–vis absorption of DBT and NR was negligible; consequently, no CD signal transfer from the chiral donor to the achiral acceptor dyes was detected.

**FIGURE 4:**
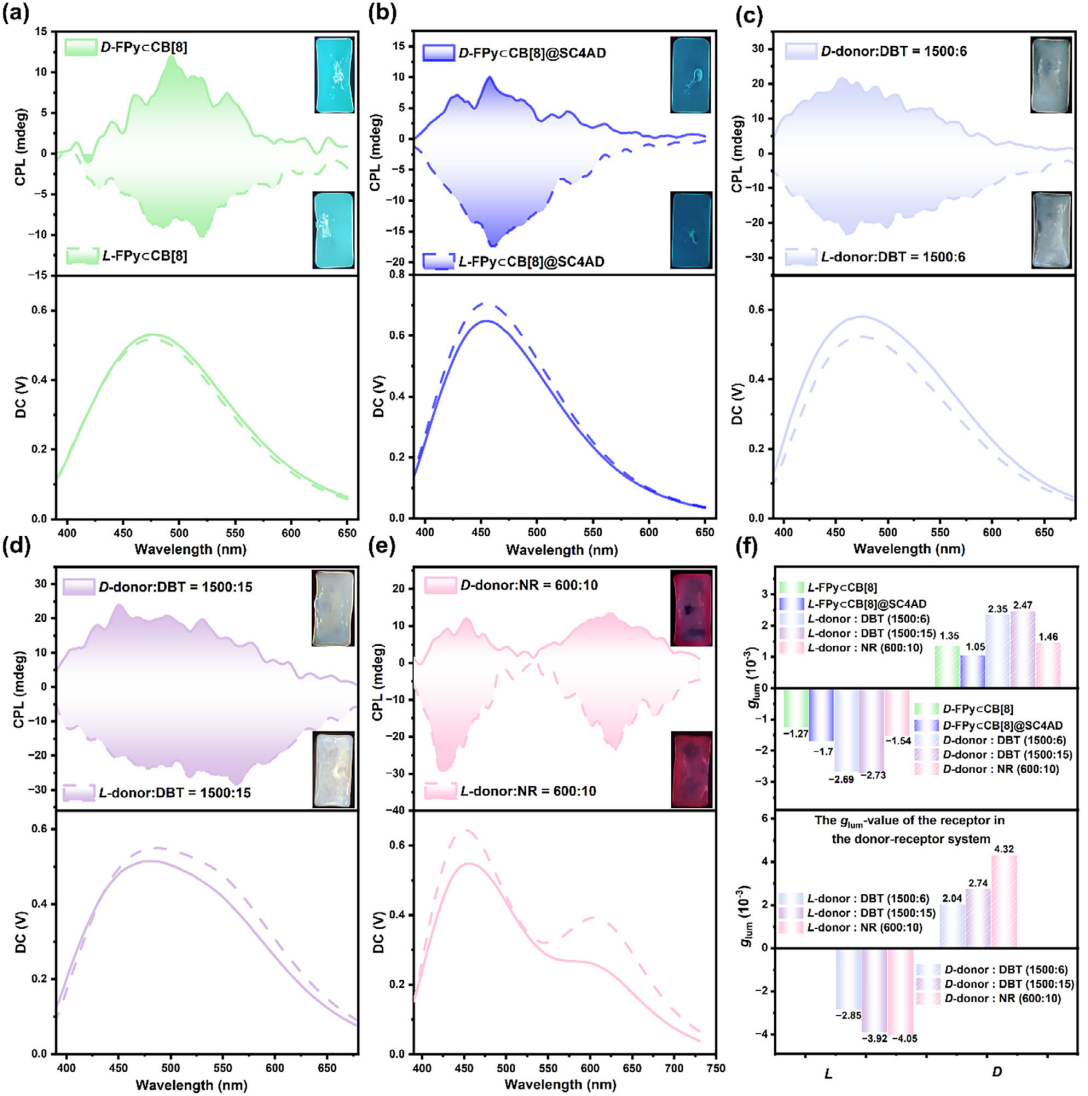
The CPL spectra and inset: photographs of the solid films of (a) *L*/*D*‐FPy⊂CB[8]@PVA, (b) *L*/*D*‐FPy⊂CB[8]@SC4AD@PVA, (c,d) *L*/*D*‐FPy⊂CB[8]@SC4AD:DBT (1500:6 and 1500:15)@PVA, (e) *L*/*D‐*FPy⊂CB[8]@SC4AD:NR@PVA. (f) The g_lum_ values of a‐e).

This study systematically investigated the construction of supramolecular chiral assemblies based on cucurbit[8]uril (CB[8]) host‐guest complexes and the precise modulation of their CPL properties. As shown in Figure S60, all assemblies displayed intense mirror‐symmetric CPL signals, confirming the successful preparation of CPL‐active systems with diverse emission colors. In contrast, studies on the monomers and assemblies formed in the absence of CB[8] revealed no mirror‐symmetric CPL signals (Figure S61). This further verifies that the charge‐transfer complexes formed by the macrocycle‐confined guest molecules within the CB[8] cavity are responsible for enabling chiral transfer and CPL generation. As indicated in Figure S60f, the *g*_lum_ of the assemblies in solution was on the order of only ∼10^−^^4^. This relatively low value suggests that dynamic disorder in the solution phase weakens the average chiroptical activity. To enhance CPL signals by improving structural order, we incorporated the assemblies into ordered polyvinyl alcohol (PVA) film matrices and re‐evaluated their CPL spectra (Figure 4). The *L*/*D*‐FPy⊂CB[8] binary inclusion complex exhibited characteristic CPL emission at 510 nm (Figure 4a). Upon co‐assembly with the amphiphilic molecule SC4AD via hydrophobic interactions and polymer confinement to form the ternary supramolecular assembly *L*/*D*‐FPy⊂CB[8]@SC4AD (Figure 4b), nonradiative transitions of the fluorophore *L*/*D*‐FPy were effectively suppressed. This process likely amplifies the chiral signal by constructing a more ordered chiral superstructure, thereby enhancing CPL performance and providing a platform for transferring chirality to achiral dyes. To further investigate CPL modulation via energy transfer, two fluorescent acceptors, DBT and NR, were introduced into the ternary assembly. Efficient chiral energy transfer from the *L*/*D*‐FPy donor to DBT and NR was demonstrated in the *L*/*D*‐FPy⊂CB[8]@SC4AD:DBT (Figure 4c,d) and *L*/*D*‐FPy⊂CB[8]@SC4AD:NR (Figure 4e) systems, respectively. The CPL emission peaks red‐shifted to the characteristic emission wavelengths of DBT and NR (approximately 560 and 640 nm). The composite films successfully retained the donor's chirality, as evidenced by an unchanged sign but a significantly altered magnitude of the *g*_lum_ factor, confirming effective chirality transfer during the energy transfer process. All composite films showed intense mirror‐symmetric CPL signals, indicating the successful fabrication of multicolor CPL‐active films. The *g*_lum_ values of these chiral fluorescent composite films were measured, with results summarized in Figure 4f. Among them, *L*/*D*‐FPy⊂CB[8]@SC4AD:NR were estimated to be − 4.05 × 10^−3^ and + 4.32 × 10^−3^, respectively, representing an order‐of‐magnitude improvement over the solution phase. The PVA matrix not only enhances the *g*_lum_ value by immobilizing the assemblies and suppressing dynamic disorder in solution, but may also further promote the formation of more ordered chiral superstructures through interactions such as hydrogen bonding, thereby amplifying the chiroptical signals [54, 55]. In summary, the evolution of CPL spectra and *g*_lum_ factors in this series clearly reveals a stepwise integration process from host‐guest complexation and ternary co‐assembly to chiral energy transfer. This work successfully demonstrates how a supramolecular hierarchical assembly strategy can be used to dynamically and precisely modulate both the intensity and wavelength of CPL. It provides a robust molecular design strategy and offers fundamental insights for developing high‐performance, customizable circularly polarized luminescent materials.

**FIGURE 5:**
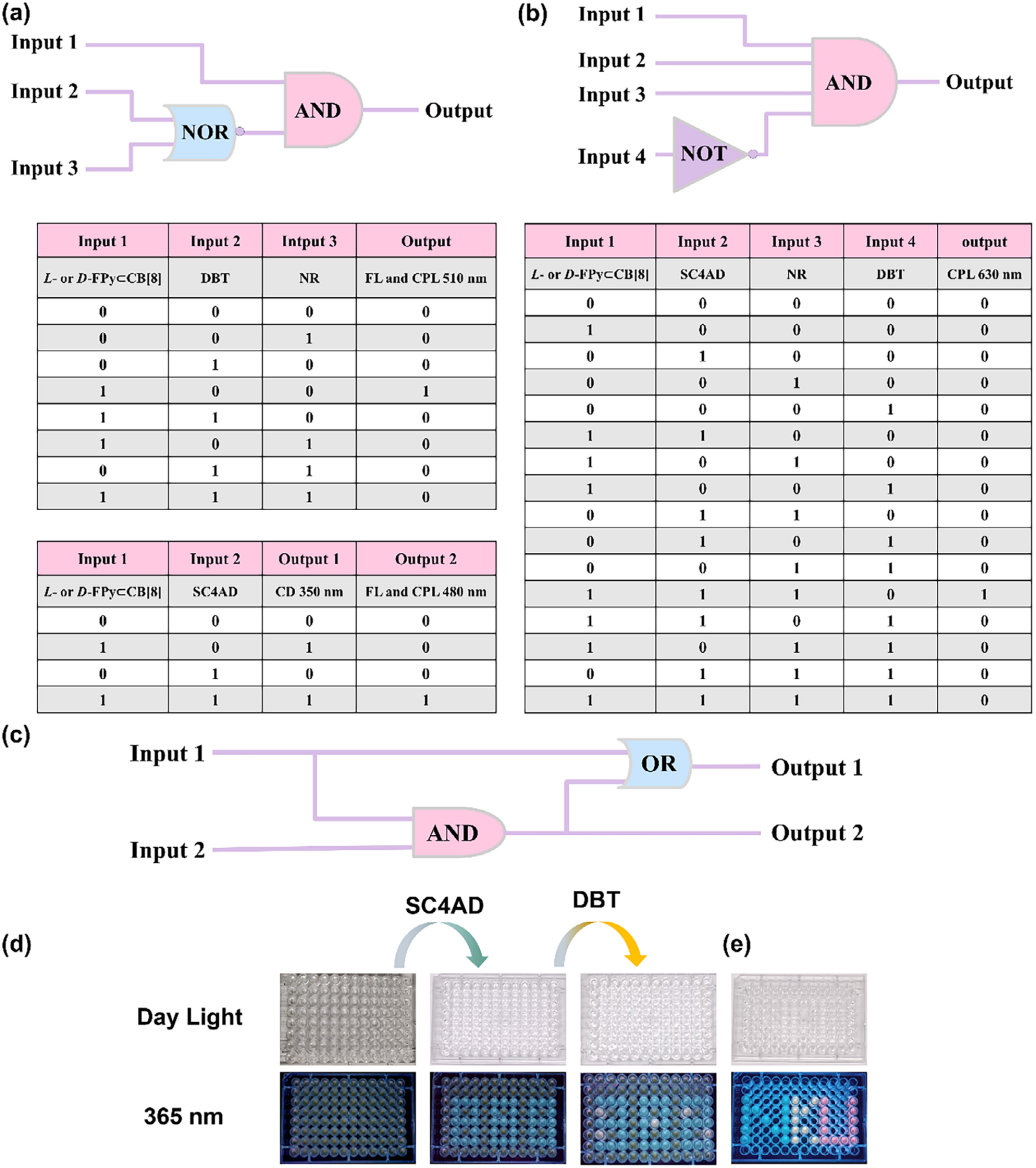
Schematic diagram and corresponding results for constructing the chiral logic gate containing (a) “NOR” gate, (b) “NOT” gate, and (c) “OR” gate. (d,e) The process of information storage using *L*/*D*‐FPy⊂CB[8], *L*/*D*‐FPy⊂CB[8]@SC4AD, *L*/*D*‐FPy⊂CB[8]@SC4AD:DBT and *L*/*D*‐FPy⊂CB[8]@SC4AD:NR.

The prepared single‐molecule chiral transfer cascade supramolecular assemblies exhibit tunable chirality and multicolor luminescence, prompting an exploration of their potential applications in chiral logic gates and information encryption.​ As shown in Figure 5, the CD signals and luminescent properties were defined as the “output”, while the conditions of *L*‐ or *D*‐FPy⊂CB[8], SC4AD, DBT, and NR were defined as the “input”. Luminescence at 510 nm was defined as “1” and its absence as “0”. When *L*‐ or *D*‐FPy⊂CB[8] was used as the input, the luminescence output was “locked”. Using both DBT and NR as inputs for a “**NOR**” gate resulted in a “silent” output signal (Figure 5a). Additionally, the co‐assembly system formed by SC4AD and *L*‐ or *D*‐FPy⊂CB[8] not only enhanced the luminescence intensity but also achieved multicolor emission. In the presence of *L*‐ or *D*‐FPy⊂CB[8], SC4AD, and NR, the red CPL output was “locked”, whereas using DBT as the input for a “**NOT**” gate yielded a “silent” output signal (Figure 5b). Figure 5c illustrates that to obtain both FL and CPL at 480 nm, both input 1 and input 2 must be set to “1” (an “AND gate”). However, to achieve a CD signal at 350 nm required only input 1 to be true, regardless of the state of input 2, corresponding to an “OR” gate. Owing to the multicolor luminescence of the supramolecular assemblies, which could be modulated by DBT and NR, they were further applied to information encryption. As depicted in Figure 5d, an aqueous solution of *L*‐ or *D*‐FPy⊂CB[8] was added dropwise to the background in the 96‐well plates. Subsequently, the addition of an SC4AD solution revealed the number “888” with blue fluorescence. Further addition of a DBT solution, then disclosed the stored information “302”. By utilizing assembly solutions of different colors, the letters N, K, and U appeared indistinguishable under daylight but became clearly readable under 365 nm irradiation (Figure 5e).

## 3 Conclusion

In summary, we constructed a cascade supramolecular assembly system for single‐molecule chiral transfer enhancement multicolor CPL based on *L*/*D*‐FPy, CB[8], SC4AD, DBT, and NR through supramolecular non‐covalent interactions. The CB[8] encapsulated *L*/*D*‐FPy to form a host‐guest complex, resulting in a bathochromic shift of approximately 60 nm with green fluorescence emission. To prevent quenching of the phosphor by water molecules, the complex was further assembled with SC4AD into nanoparticles via electrostatic interactions, leading to an approximately 360‐fold enhancement in fluorescence intensity. Co‐assembly of the *L*/*D*‐FPy⊂CB[8]@SC4AD system with polyvinyl alcohol (PVA) into a solid film further enhanced the circularly polarized luminescence behavior, demonstrating that the assemblies exhibit intense mirror‐image circularly polarized luminescence signals and successfully transfer chirality to achiral dyes with luminescence asymmetry factor values for *L*/*D*‐FPy⊂CB[8]@SC4AD:NR were estimated to be −4.05 × 10^−3^ and + 4.32 × 10^−3^, respectively, which also successfully applied in information encryption and chiral logic gates. Overall, the assembly provides an effective approach for cascading enhancement of chiral transfer and circularly polarized luminescence.

## Funding

This work was supported by the National Nature Science Foundation of China (NNSFC, Grant Nos. 22131008, 22361036, 22571173), Fundamental Research Funds for the Central Universities (Nankai University), and Inner Mongolia Natural Science Excellent Youth Foundation No. 2025YQ050).

## Conflicts of Interest

The authors declare no conflicts of interest.

## Supporting information




**Supporting File**: advs75360‐sup‐0001‐SuppMat.docx.

## Data Availability

The data that support the findings of this study are available from the corresponding author upon reasonable request.
