# Supplementary material for: Cucurbit[8]Uril Achieved Single‐Molecule Chiral Transfer to Supramolecular Circularly Polarized Luminescence
Source: Adv Sci (Weinh). 2026 Apr 20;13(39):e75360. doi: 10.1002/advs.75360 (PMC13335517; doi:10.1002/advs.75360)
Supplement: Supplementary file 1 — Supporting File: advs75360‐sup‐0001‐SuppMat.docx. [file ADVS-13-e75360-s001.docx]

Supporting Information

**Cucurbit[8]uril Achieved Single-Molecule Chiral Transfer to Supramolecular Circularly Polarized Luminescence**

Yongxue Li^[a]^, Jie Wu^[a]^, Weiheng Zhang^[a]^, Hengzhi Zhang^[a]^, Wei-Lei Zhou*^[a], [b]^, and Yu Liu*^[a]^

[a] *College of Chemistry, State Key Laboratory of Elemento-Organic Chemistry,*

*Nankai University, Tianjin 300071, P. R. China*

[b] *College of Chemistry and Material Science, The Technological Innovation Center of Supramolecular Chinese (Mongolian) Medicine*

*Inner Mongolia Minzu University, Tongliao 028000, P. R. China*

* Corresponding author:

E-mail: zhouweilei_2011@163.com

E-mail: yuliu@nankai.edu.cn

**Table of Contents**

[1. Instruments and Methods 3](#_Toc214030475)

[2. Synthesis and Characterization of Compounds 5](#_Toc214030476)

[3. Characterization and Photophysical Properties of Assemblies. 24](#_Toc214030477)

[4. Reference 37](#_Toc214030478)

**Experimental Section**

# Instruments and Methods

All the reagents and solvents were commercially available and used as received without further purification. Column chromatography was performed on silica gel (100-200 mesh). NMR spectra were recorded on a Bruker AV400 instrument at 298 K, and chemical shifts were recorded in parts per million (ppm). 2D COSY and NOESY spectra were measured on a Zhongke-Oxford I-400 instrument. High-resolution mass spectra (HR-MS) were measured on an ESI-TOF-HRMS and AB Sciex Triple TOF 5600+. UV-vis spectra were recorded on a Shimadzu UV-3600 spectrophotometer equipped with a PTC-348WI temperature controller. The photoluminescence spectra were measured by time-correlated single-photon counting on an FLS980 instrument (Edinburgh Instruments Ltd., Livingston, UK). The lifetimes and quantum yield were recorded by the FS5 instrument (Edinburgh Instruments, Livingston, UK). The CPL spectra were performed on the JASCO CPL-300. The samples for transmission electron microscope (TEM) measurements were prepared by dropping the solution onto copper grids and then air-drying. Geometry optimization of *L*/*D*-FPy and supramolecular assembly *L*/*D*-FPy⸦CB[8] were performed in Gaussian 16.

**1.1 Preparation of multicolor PVA films**

**10% PVA:** 10 g PVA was added to 100 mL deionized water and stirred at 100°C for 5 h^[1]^.

**SC4AD solution:** SC4AD (12.82 mg, 0.01 mmol) was added to dimethyl sulfoxide (2 mL) and sonicated for 30 min.

**Preparation of supramolecular assembly of *L*-FPy⊂CB[8]**^[2]^**:** *L*-FPy (4.58 mg, 0.01 mmol), CB[8] (13.29 mg, 0.01 mmol) were added to water (2 mL) and sonicated for one day.

**Preparation supramolecular films of *L*-FPy⊂CB[8]:**

A mixed solution of *L*-FPy⊂CB[8] (200 μL), dimethyl sulfoxide (100 μL), and 10% PVA water solution (200 μL) was added. The mixture was sonicated for 30 min. Then, 0.3 ml of the resulting mixture was dropped onto quartz glass slides using a syringe and heated until all the solvent had evaporated.

**Preparation of supramolecular films of *L*-FPy⊂CB[8]@SC4AD:** A mixed solution of *L*-FPy⊂CB[8] (200 μL), SC4AD (100 μL), and 10% PVA water solution (200 μL) was added. The mixture was sonicated for 30 min. Then, 0.3 ml of the resulting mixture was dropped onto quartz glass slides using a syringe and heated until all the solvent had evaporated.

**Preparation of supramolecular films of *L*-FPy⊂CB[8]@SC4AD:DBT@PVA and *L*-FPy⊂CB[8]@SC4AD:NR@PVA:** A mixed solution of *L*-FPy⊂CB[8] (200 μL), SC4AD (100 μL), add dye molecules (DBT or NR) according to different molar ratios, and 10% PVA water solution (200 μL) was added. The mixture was sonicated for 30 min. Then, 0.3 ml of the resulting mixture was dropped onto quartz glass slides using a syringe and heated until all the solvent had evaporated.

**Preparation of** ***D*- supramolecular assembly films:** This is similar to what has been described for *L*-supramolecular assembly films.

# Synthesis and Characterization of Compounds


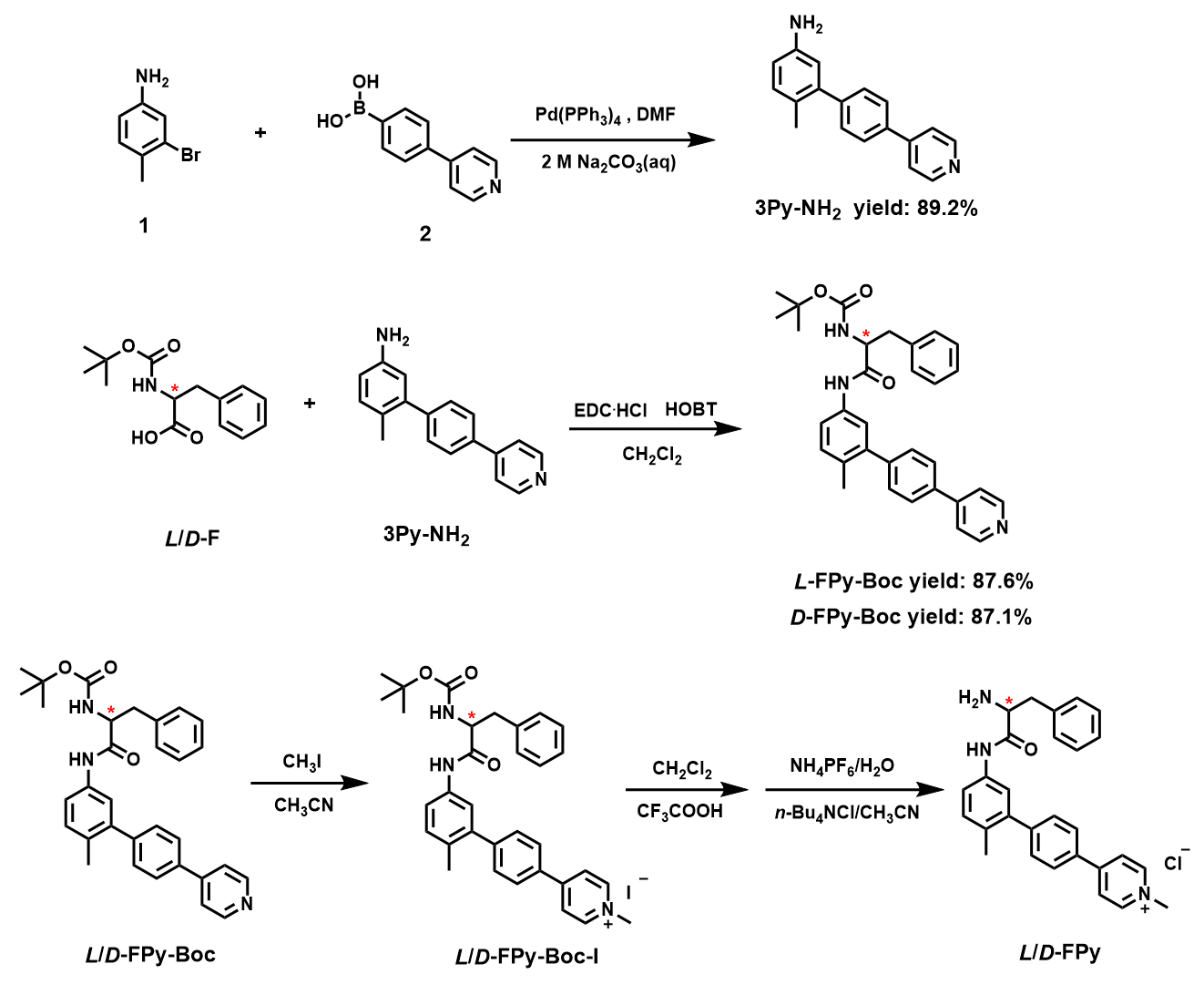


**Scheme S1**. Synthetic route of compounds.

**Compound 3Py-NH_2_:**

3Py-NH_2_ was synthesized according to the literature^[3]^. A three-necked round-bottom flask was charged with DMF (30 mL) and 2 M Na_2_CO_3_ solution (10 mL). And the 4-Pyridineboronic acid (1.612 g, 8.1 mmol), 3-bromo-4-methylaniline (1.00 g, 5.4 mmol) were added to the solution and degassed for 10 min. Tetrakis(triphenylphosphine)palladium(0) (0.44 g, 0.38 mmol) was added to the solution and degassed for 30 min. The mixture was heated under reflux overnight. The reaction solution was diluted with H_2_O (10 mL), the crude was dissolved in CH_2_Cl_2,_ and was extracted three times with water. The combined organic layer was dried with anhydrous Na_2_SO_4_, filtered, and the solvent was removed by evaporation. The resulting residue was further purified by column chromatography (petroleum ether/ethyl acetate = 3:1), and 3Py-NH_2_ was obtained as a pale-yellow solid (1.25 g, 89.2%).

^1^H NMR (400 MHz, DMSO-*d_6_*, 298 K, ppm) δ 8.64 (d, *J* = 6.2 Hz, 2H), 7.85 (d, *J* = 8.3 Hz, 2H), 7.75 (d, *J* = 6.2 Hz, 2H), 7.43 (d, *J* = 8.3 Hz, 2H), 6.95 (d, *J* = 8.0 Hz, 1H), 6.56-6.46 (m, 2H), 4.95 (s, 2H), 2.09 (s, 2H).

^13^C NMR (100 MHz, DMSO-*d_6_*, 298 K, ppm) δ 150.73, 147.13, 147.08, 143.68, 141.24, 135.71, 131.40, 130.08, 126.99, 121.76, 121.55, 115.62, 114.00, 19.66.

HRMS (ESI) m/z for 3Py-NH_2_ [C_18_H_16_N_2_] calcd. for [M + H]^+^ 261.13862, found: 261.13860.

**Figure S1**. ^1^H NMR spectrum of compound 3Py-NH_2_ (400 MHz, DMSO-*d_6_*, 298 K).

**Figure S2**. ^13^C NMR spectrum of compound 3Py-NH_2_ (100 MHz, DMSO-*d_6_*, 298 K).


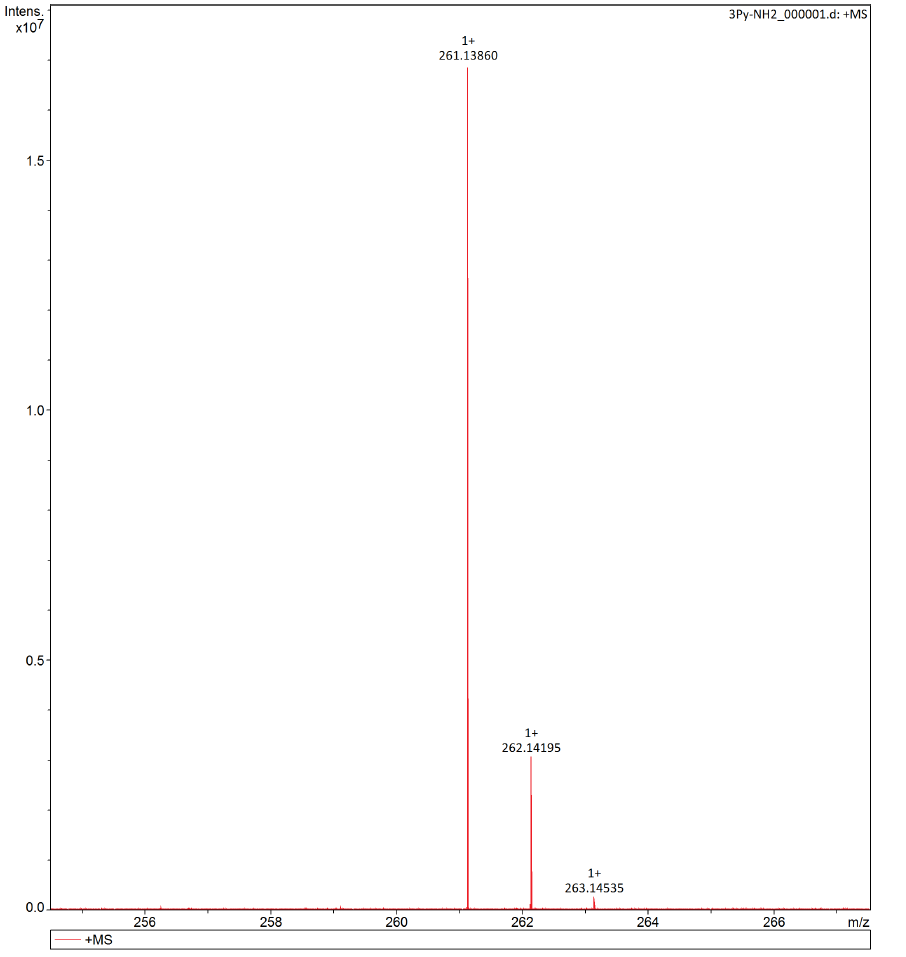


**Figure S3**. HR-MS spectrum of 3Py-NH_2_.

**Compound *L*-FPy-Boc:**

A solution of 3Py-NH_2_ (260.3 mg, 1.0 mmol), (tert-butoxycarbonyl)-*L*-phenylalanine (318.4 mg, 1.2 mmol), 1-(3-dimethylaminopropyl)-3-ethylcarbodiimide hydrochloride (287.6 mg, 1.5 mmol), and 1-Hydroxybenzotriazole (202.7 mg, 1.5 mmol) in 40 mL CH_2_Cl_2_ (ice bath) for overnight. After the reaction was finished, the reaction mixture was extracted three times with 1 M HCl aqueous solution and CH_2_Cl_2_, and the organic phases were combined. The organic phase was then extracted once with saturated NaHCO_3_. The combined organic layer was dried with anhydrous Na_2_SO_4_, filtered, and the solvent was removed by evaporation. The resulting residue was further purified by column chromatography (dichloromethane/methanol = 50:1), *L*-FPy-Boc was obtained as a light yellow crystalline solid (444.9 mg, 87.6%).

^1^H NMR (400 MHz, DMSO-*d_6_*, 298 K, ppm) δ 10.03 (s, 1H), 8.66 (d, *J* = 6.1 Hz, 2H), 7.91 (d, *J* = 8.0 Hz, 2H), 7.78 (d, *J* = 6.2 Hz, 2H), 7.53-7.49 (m, 4H), 7.32-7.25 (m, 5H), 7.20 (d, *J* = 6.9 Hz, 1H), 7.10 (d, *J* = 8.3 Hz, 1H), 4.31 (s, 1H), 3.01-2.97 (m, 1H), 2.91-2.73 (m, 1H), 2.22 (s, 3H), 1.31 (s, 9H).

^13^C NMR (100 MHz, DMSO-*d_6_*, 298 K, ppm) δ 171.17, 155.85, 150.76, 147.01, 142.65, 141.13, 138.43, 137.39, 131.20,136.19, 130.16, 130.04, 129.71, 128.51, 127.21, 126.75, 121.61, 120.81, 119.03, 78.55, 57.04, 37.97, 28.62, 20.05.

HRMS (ESI) m/z for *L*-FPy-Boc [C_32_H_33_N_3_O_3_] calcd. for [M + Na]^+^ 530.24141, found: 530.24154.

**Figure S4**. ^1^H NMR spectrum of compound *L*-FPy-Boc. (400 MHz, DMSO-*d_6_*, 298 K).

**Figure S5**. ^13^C NMR spectrum of compound *L*-FPy-Boc (100 MHz, DMSO-*d_6_*, 298 K).


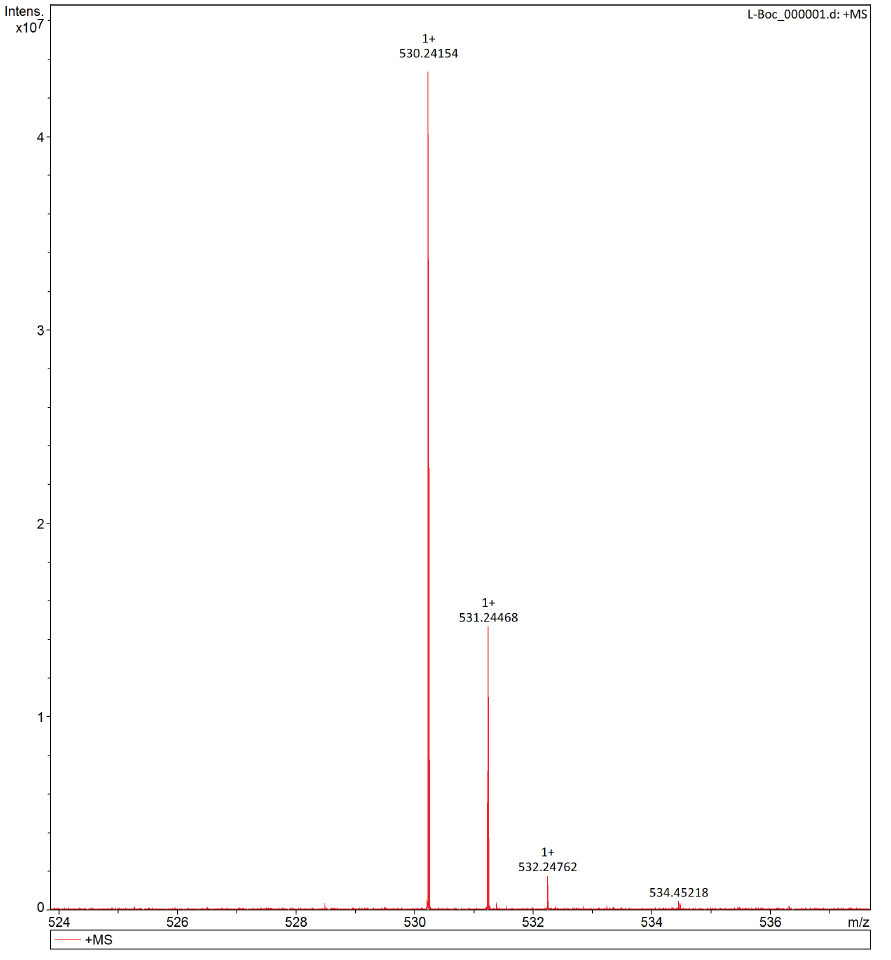


**Figure S6**. HR-MS spectrum of *L*-FPy-Boc.

**Compound *D*-FPy-Boc:**

Similar to that described for compound *L*-FPy-Boc (442.1 mg, 87.1%).

^1^H NMR (400 MHz, DMSO-*d_6_*, 298 K, ppm) δ 10.03 (s, 1H), 8.66 (d, *J* = 6.2 Hz, 2H), 7.90 (d, *J* = 8.0 Hz, 2H), 7.78 (d, *J* = 6.2 Hz, 2H), 7.57-7.45 (m, 4H), 7.36-7.24 (m, 5H), 7.20 (d, *J* = 7.0 Hz, 1H), 7.10 (d, *J* = 8.2 Hz, 1H), 4.31 (s, 1H), 3.01-2.97 (m, 1H), 2.89-2.77 (m, 1H), 2.22 (s, 3H), 1.31 (s, 9H).

^13^C NMR (100 MHz, DMSO-*d_6_*, 298 K, ppm) δ 171.17, 155.85, 150.75, 147.01, 142.65, 141.13, 138.43, 137.39, 131.21,136.19, 130.16, 130.04, 129.71, 128.51, 127.20, 126.75, 121.61, 120.82, 119.04, 78.55, 57.05, 37.98, 28.63, 20.06.

HRMS (ESI) m/z for *D*-FPy-Boc [C_32_H_33_N_3_O_3_] calcd. for [M + Na]^+^ 530.24141, found: 530.24143.

**Figure S7**. ^1^H NMR spectrum of compound *D*-FPy-Boc. (400 MHz, DMSO-*d_6_*, 298 K).

**Figure S8**. ^13^C NMR spectrum of compound *D*-FPy-Boc (100 MHz, DMSO-*d_6_*, 298 K).


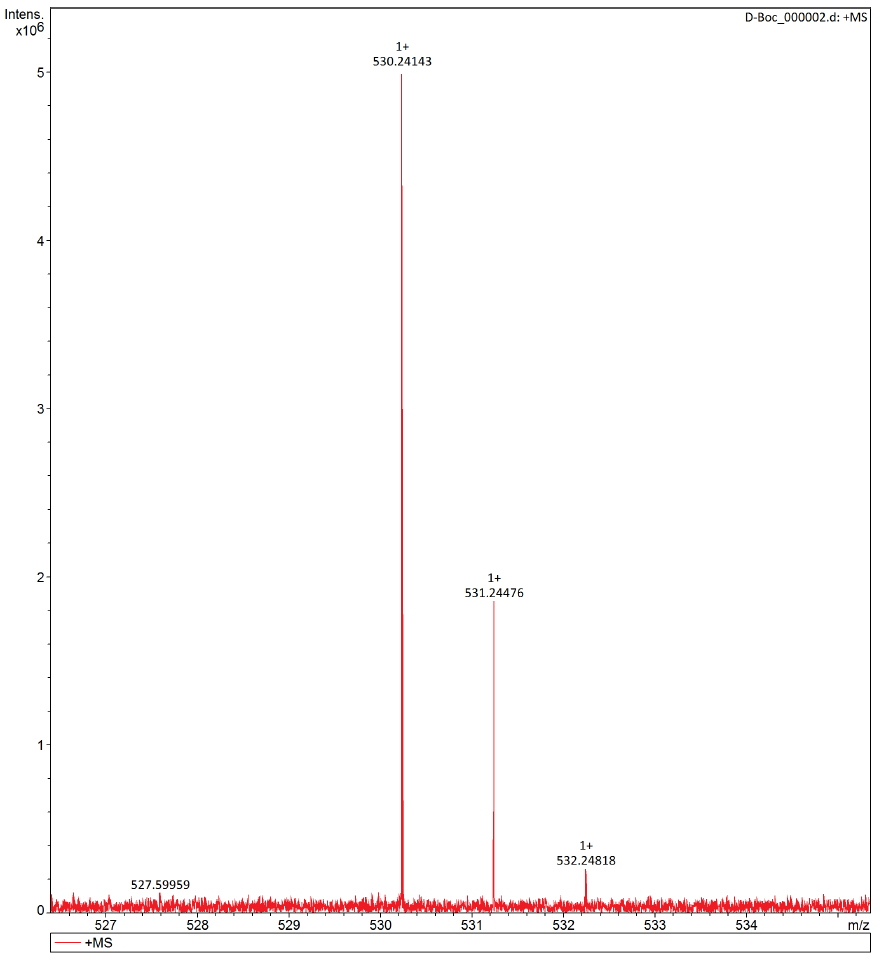


**Figure S9**. HR-MS spectrum of *D*-FPy-Boc.

**Compound *L*-FPy-Boc-I:**

*L*-FPy-Boc (152.3 mg, 0.3 mmol) was dispersed in 30 mL anhydrous acetonitrile under an argon atmosphere. Then, methyl iodide (1.8 mL, 30 mmol, 100 eq) was added to the system by injection. The mixture was stirred for 24 h under reflux conditions. After the reaction was finished, the mixture was cooled to room temperature and then filtered to collect the solid. The solid was washed with acetonitrile and dried in a vacuum at 50°C overnight to afford the product with counterion ion I^－^ as a yellow solid (171.6 mg, 88.1%).

^1^H NMR (400 MHz, DMSO-*d_6_*, 298 K, ppm) δ 10.06 (s, 1H), 9.03 (d, *J* = 6.4 Hz, 2H), 8.57 (d, *J* = 6.7 Hz, 2H), 8.18 (d, *J* = 8.1 Hz, 2H), 7.62 (d, *J* = 8.0 Hz, 2H), 7.59 (d, *J* = 2.2 Hz, 1H), 7.50 (d, *J* = 8.0 Hz, 1H), 7.33-7.26 (m, 5H), 7.20 (t, *J* = 6.9 Hz, 1H), 7.10 (d, *J* = 8.2 Hz, 1H), 4.35 (s, 4H), 3.02-2.97 (m, 1H), 2.91-2.76 (m, 1H), 2.23 (s, 3H), 1.31 (s, 9H).

^13^C NMR (100 MHz, DMSO-d6, 298 K, ppm) δ 171.23, 155.85, 154.32, 146.07, 145.44, 140.57, 138.39, 137.46, 132.61, 131.35, 130.61, 130.04, 129.71, 128.57, 128.52, 126.77, 124.47, 120.70, 119.40, 78.58, 57.04, 47.59, 37.94, 28.63, 20.02.

**Figure S10**. ^1^H NMR spectrum of compound *L*-FPy-Boc-I. (400 MHz, DMSO-*d_6_*, 298 K).

**Figure S11**. ^13^C NMR spectrum of compound *L*-FPy-Boc-I. (100 MHz, DMSO-*d_6_*, 298 K).

**Compound *D*-FPy-Boc-I:**

Similar to that described for compound *L*-FPy-Boc-I (170.3 mg, 87.4%).

^1^H NMR (400 MHz, DMSO-d6, 298 K, ppm) δ 10.06 (s, 1H), 9.03 (d, *J* = 6.4 Hz, 2H), 8.57 (d, *J* = 6.7 Hz, 2H), 8.18 (d, *J* = 8.1 Hz, 2H), 7.62 (d, *J* = 8.2 Hz, 2H), 7.59 (d, *J* = 2.2 Hz, 1H), 7.50 (d, *J* = 7.7 Hz, 1H), 7.30 (m, 5H), 7.20 (t, *J* = 7.0 Hz, 1H), 7.09 (d, *J* = 8.3 Hz, 1H), 4.35 (s, 4H), 3.02-2.97 (m, 1H), 2.92-2.78 (m, 1H), 2.23 (s, 3H), 1.32 (s, 9H).

^13^C NMR (100 MHz, DMSO-*d*_6_, 298 K, ppm) δ 171.23, 155.85, 154.32, 146.08, 145.44, 140.58, 138.38, 137.45, 132.62, 131.35, 130.62, 130.05, 129.70, 128.57, 128.52, 126.77, 124.47, 120.71, 119.39, 78.58, 57.04, 47.57, 37.94, 28.62, 20.02.

**Figure S12**. ^1^H NMR spectrum of compound *D*-FPy-Boc-I. (400 MHz, DMSO-*d_6_*, 298 K).

**Figure S13**. ^13^C NMR spectrum of compound *D*-FPy-Boc-I. (100 MHz, DMSO-*d_6_*, 298 K).

**Compound *L*-FPy:**

Dissolve the compound in a solvent. A mixed solution of *L*-FPy-Boc-I (130.0 mg, 0.2 mmol) in 30 mL dichloromethane and trifluoroacetic acid (*v*:*v* = 1:1) for 6 h. After the reaction was finished, and removed of the solvent was removed by evaporation, and the dry, collected solid and proceeded directly to the next step. Dispersed the solid in methanol, then slowly added saturated ammonium hexafluorophosphate aqueous solution for ion exchange, filtered to obtain a white solid, and then washed with methanol (10 mL). Subsequently, the obtained solid was dispersed in acetonitrile, and a saturated solution of tetrabutylammonium chloride in acetonitrile was slowly added to the mixture until ion exchange was complete. Filtered to collect solid and obtained white solid (76.3 mg, 83.3%).

^1^H NMR (400 MHz, D_2_O, 298 K, ppm) δ 8.78 (d, *J* = 6.5 Hz, 2H), 8.34 (d, *J* = 6.5 Hz, 2H), 8.04 (d, *J* = 8.1 Hz, 2H), 7.61 (d, *J* = 8.0 Hz, 2H), 7.41-7.31 (m, 6H), 7.20 (d, *J* = 8.6 Hz, 1H), 7.11 (d, *J* = 8.2 Hz, 1H), 4.39 (s, 3H), 4.34-4.30 (m, 1H), 3.39-3.34 (m, 1H), 3.24-3.19 (m, 1H), 2.26 (s, 3H).

^13^C NMR (100 MHz, DMSO-*d_6_*, 298 K, ppm) δ 167.27, 155.05, 144.72, 144.17, 140.37, 133.72, 133.03, 132.19, 131.34, 130.15, 129.41, 129.02, 127.89, 127.66, 124.26, 122.22, 120.96, 54.82, 47.19, 37.10, 19.16.

HRMS (ESI) m/z for *L*-FPy [C_28_H_28_ClN_3_O] calcd. For [M-Cl^－^]^＋^ 422.2227, found: 422.2230.

**Figure S14**. ^1^H NMR spectrum of compound *L*-FPy. (400 MHz, D_2_O, 298 K).

**Figure S15**. ^13^C NMR spectrum of compound *L*-FPy. (100 MHz, D_2_O, 298 K).


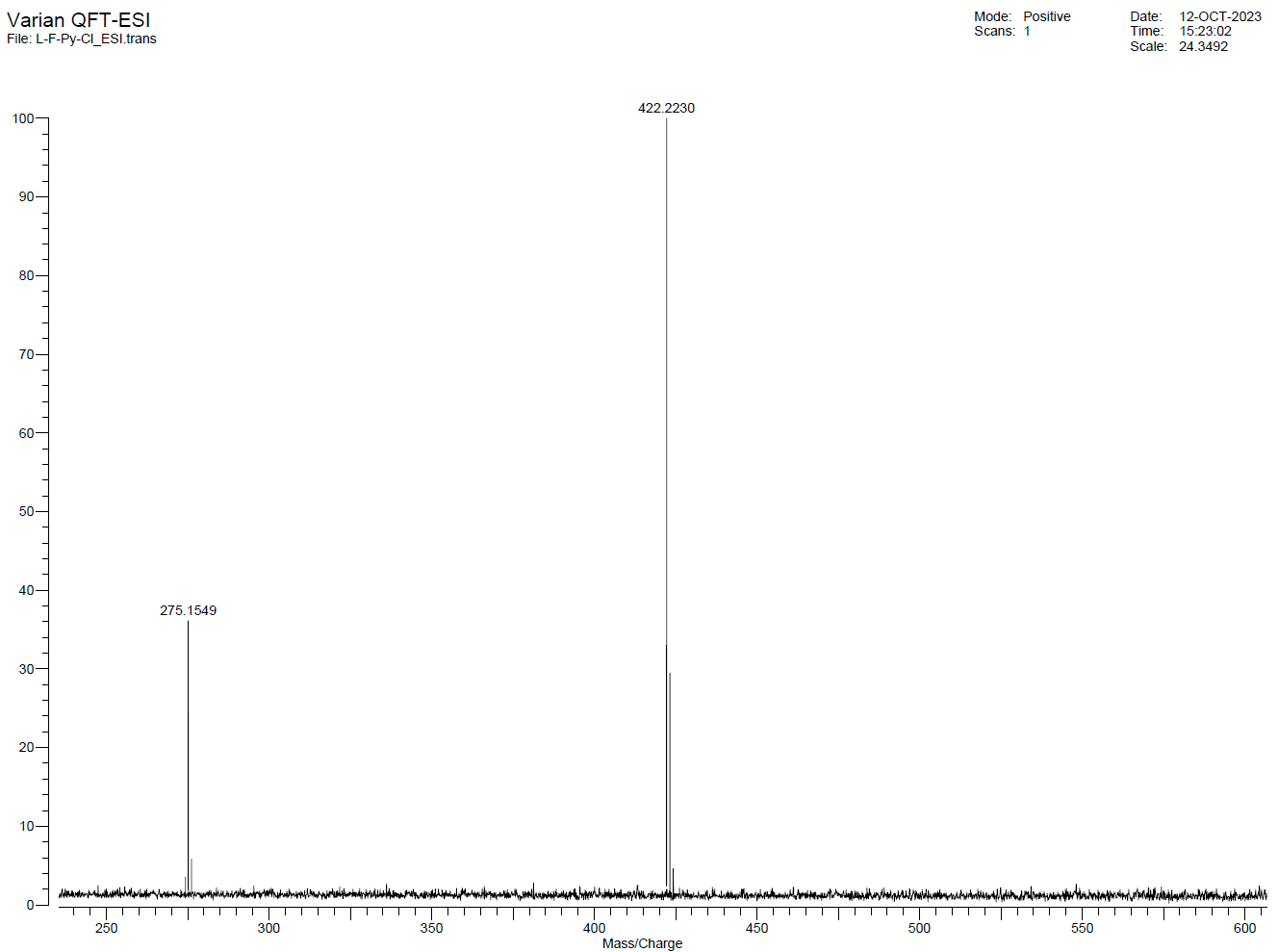


**Figure S16**. HR-MS spectrum of *L*-FPy.

**Compound *D*-FPy:**

Similar to that described for compound *L*-FPy (74.8 mg, 81.7%).

^1^H NMR (400 MHz, D_2_O, 298 K, ppm) δ 8.79 (d, *J* = 6.5 Hz, 2H), 8.35 (d, *J* = 6.5 Hz, 2H), 8.06 (d, *J* = 8.1 Hz, 2H), 7.62 (d, *J* = 8.1 Hz, 2H), 7.43-7.32 (m, 6H), 7.21 (d, *J* = 8.3 Hz, 1H), 7.12 (s, 1H), 4.40 (s, 3H), 4.35-4.31 (m, 1H), 3.41-3.36 (m, 1H), 3.28-3.18 (m, 1H), 2.28 (s, 3H).

^13^C NMR (100 MHz, DMSO-*d_6_*, 298 K, ppm) δ 167.42, 155.19, 144.75, 144.24, 140.48, 133.76, 133.67, 133.19, 132.31, 131.35, 130.19, 129.42, 129.05, 127.91, 127.72, 124.35, 122.33, 121.06, 54.87, 47.20, 37.14, 19.16.

HRMS (ESI) m/z for *D*-FPy [C_28_H_28_ClN_3_O] calcd. For [M-Cl^¯^]^＋^ 422.2227, found: 422.2231.

**Figure S17**. ^1^H NMR spectrum of compound *D*-FPy. (400 MHz, D_2_O, 298 K).

**Figure S18**. ^13^C NMR spectrum of compound *D*-FPy. (100 MHz, D_2_O, 298 K).


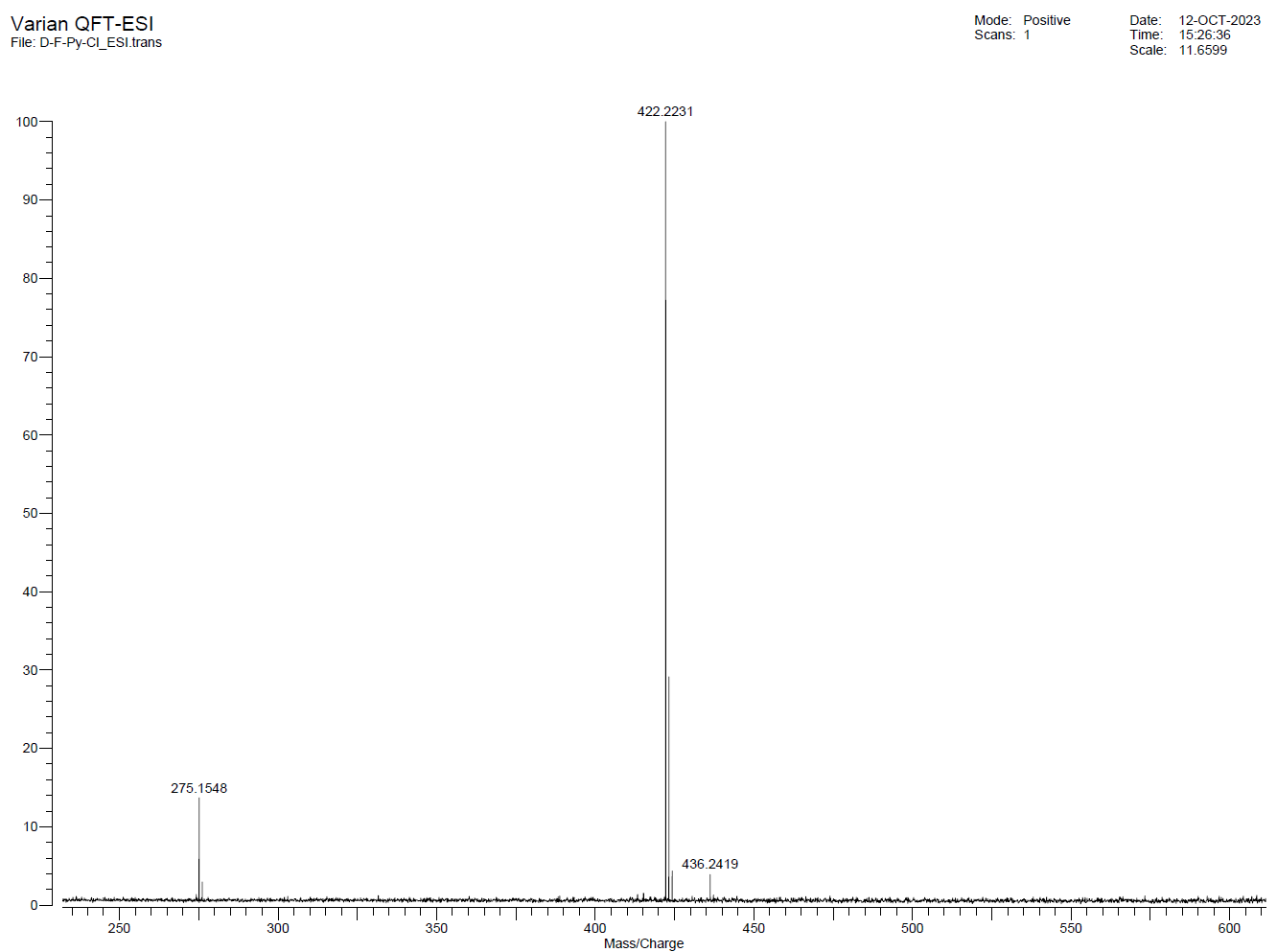


**Figure S19**. HR-MS spectrum of *D*-FPy.


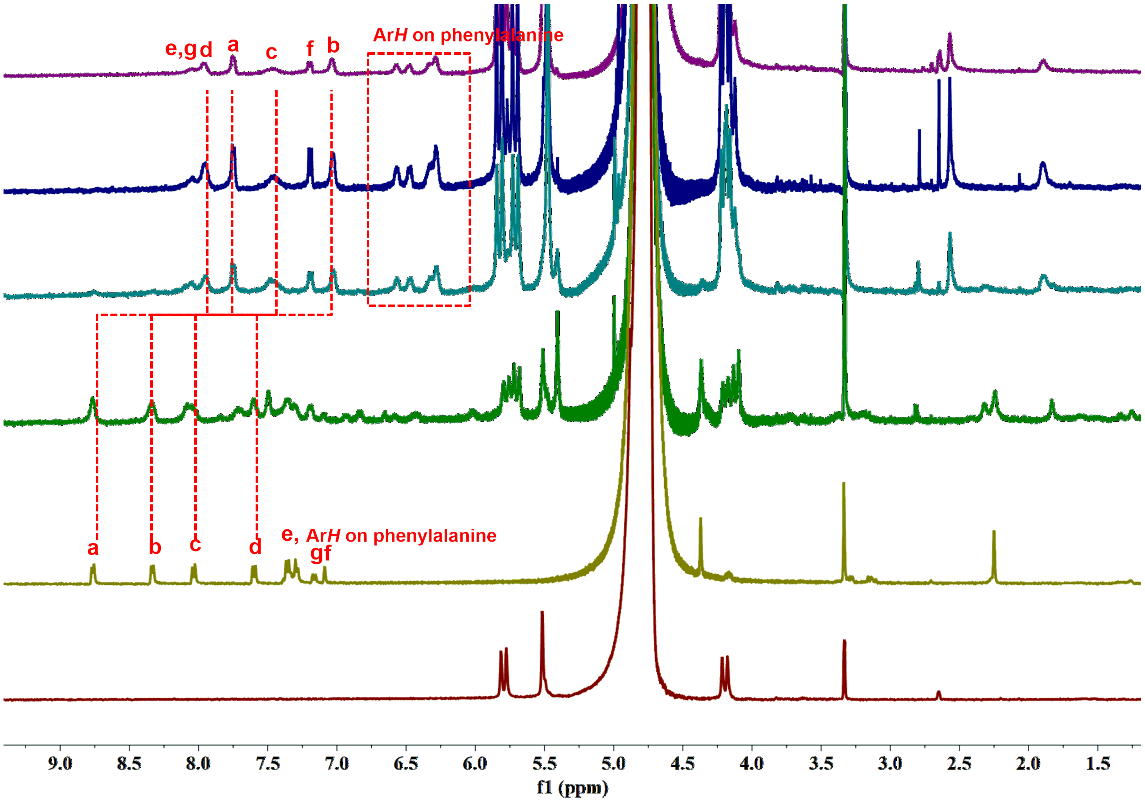


**Figure S20**. Changes of the ^1^H NMR signals of *L*-FPy (400 MHz, D_2_O, 298K) at different ratios of *L*-FPy and CB[8] ([*L*-FPy] = 5.0×10^-4^ M, [CB[8]] = 0－1.0×10^-3^ M).


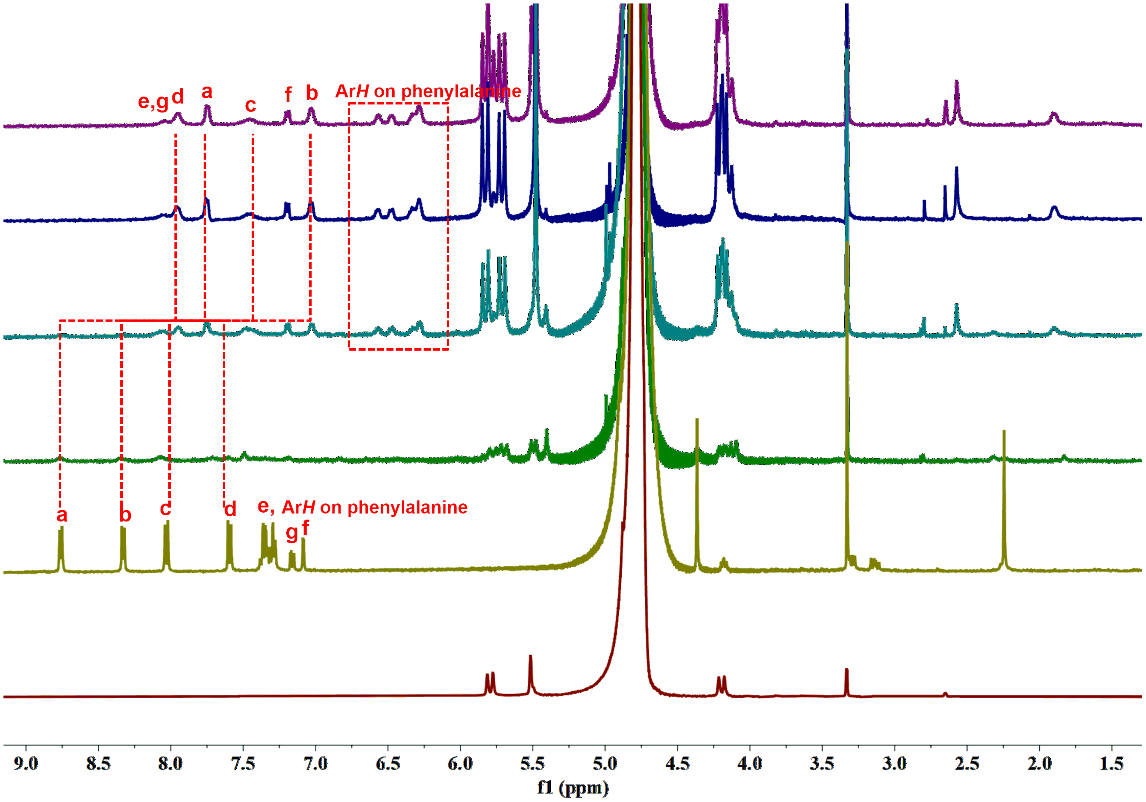


**Figure S21**. Changes of the ^1^H NMR signals of *D*-FPy (400 MHz, D_2_O, 298K) at different ratios of *D*-FPy and CB[8] ([*D*-FPy] = 5.0×10^-4^ M, [CB[8]] = 0－1.0×10^-3^ M).


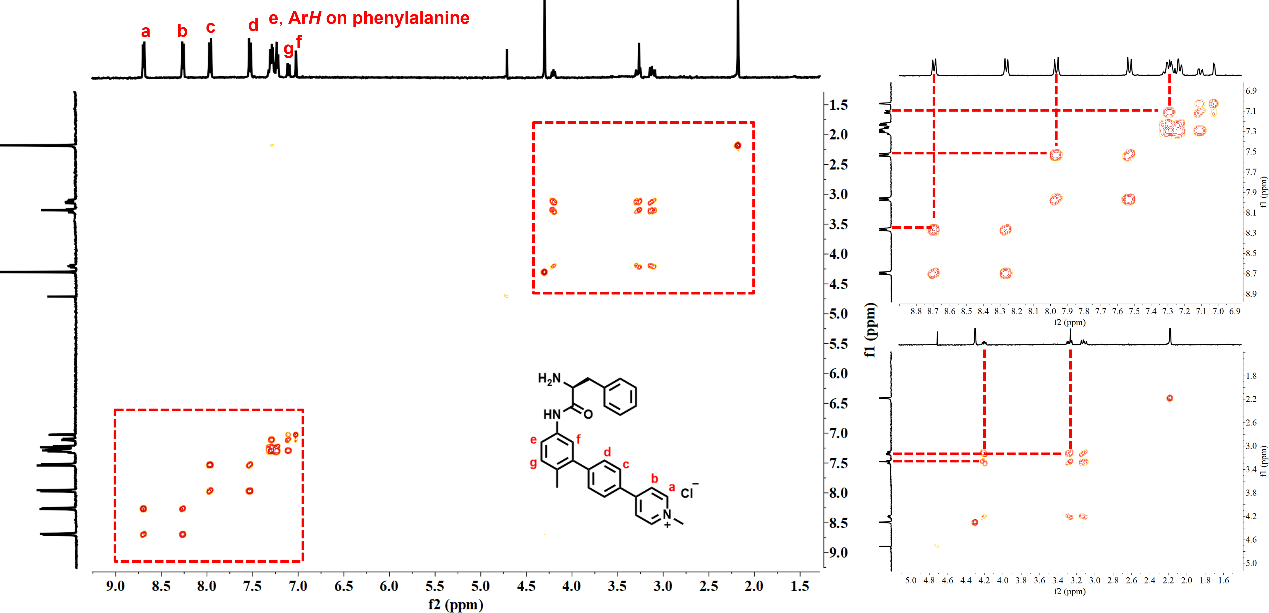


**Figure S22**. 2D ^1^H-^1^H COSY Spectrum (400 MHz, D_2_O, 298K) of *L*-FPy ([*L*-FPy] = 5.0×10^-4^ M). The right image is an enlarged version of the left image.


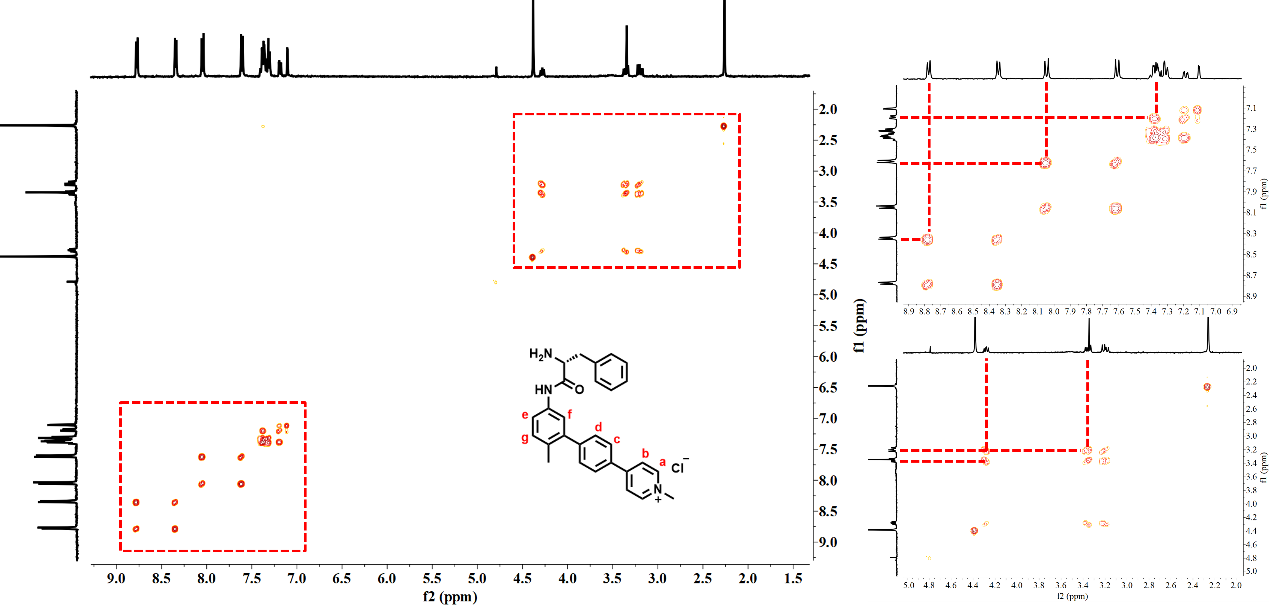


**Figure S23**. 2D ^1^H-^1^H COSY spectrum (400 MHz, D_2_O, 298K) of *D*-FPy ([*D*-FPy] = 5.0×10^-4^ M). The right image is an enlarged version of the left image.


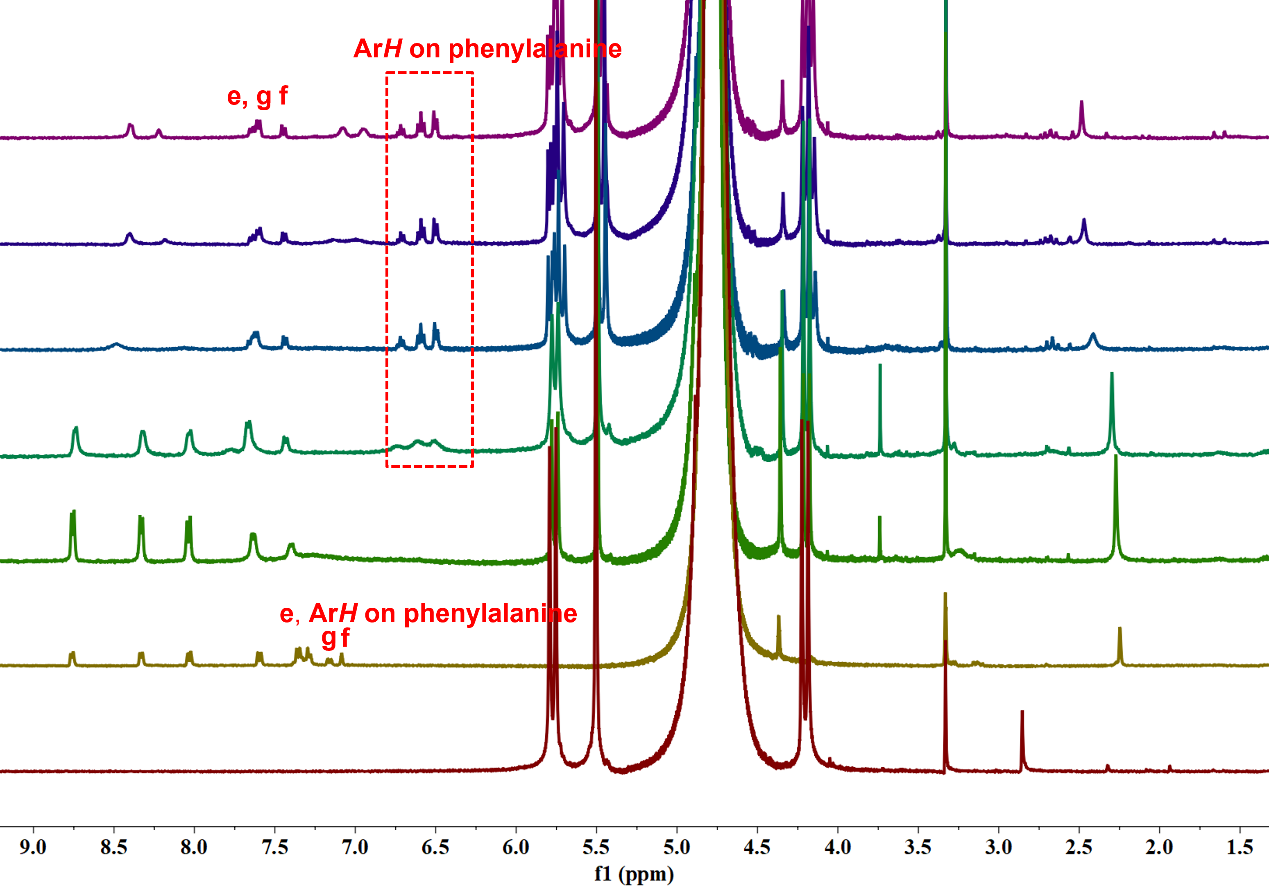


**Figure S24**. Changes of the ^1^H NMR signals of *L*-FPy (400 MHz, D_2_O, 298K) at different ratios of *L*-FPy and CB[7] ([*L*-FPy] = 5.0×10^-4^ M, [CB[7]] = 0－1.25×10^-3^ M).


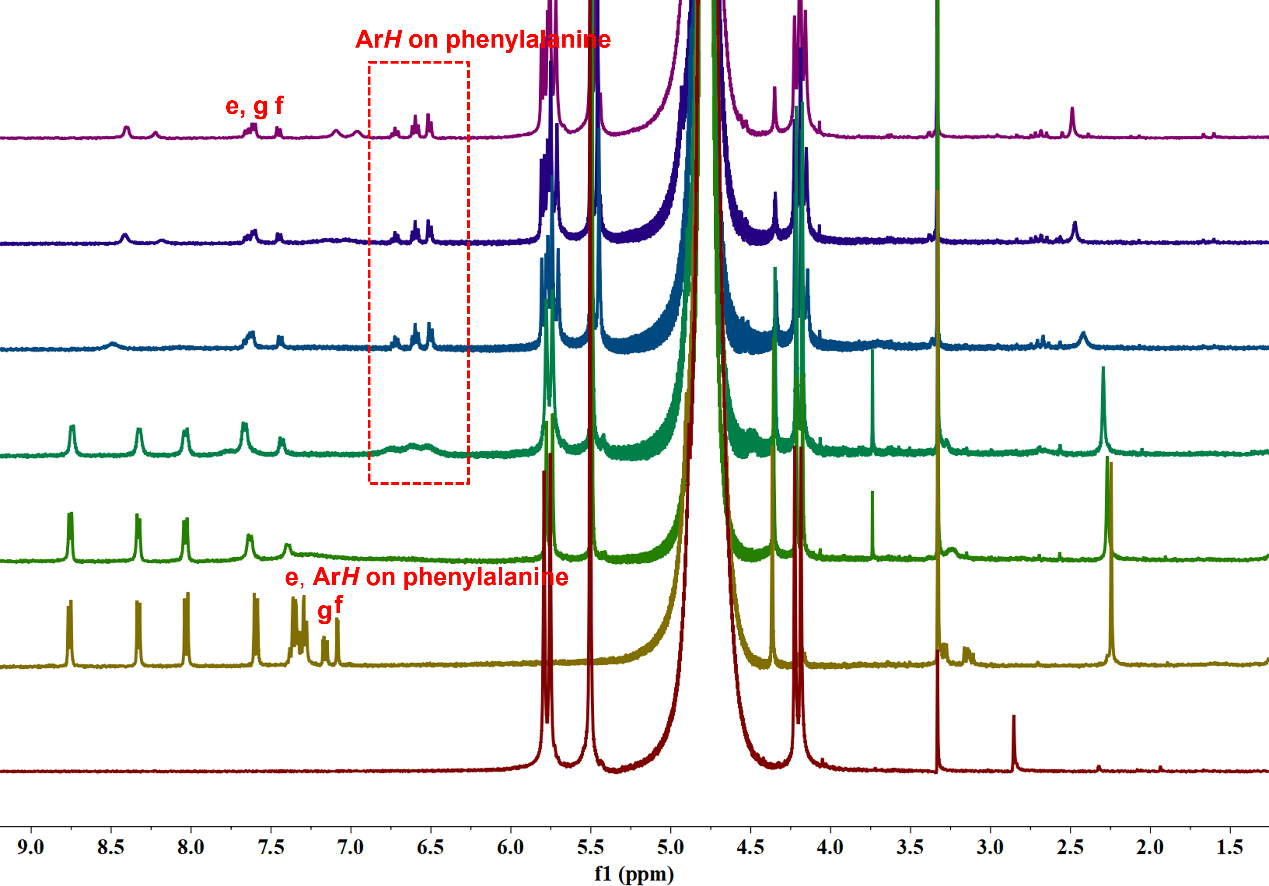


**Figure S25**. Changes of the ^1^H NMR signals of *D*-FPy (400 MHz, D_2_O, 298K) at different ratios of *D*-FPy and CB[7] ([*D*-FPy] = 5.0×10^-4^ M, [CB[7]] = 0－1.25×10^-3^ M).


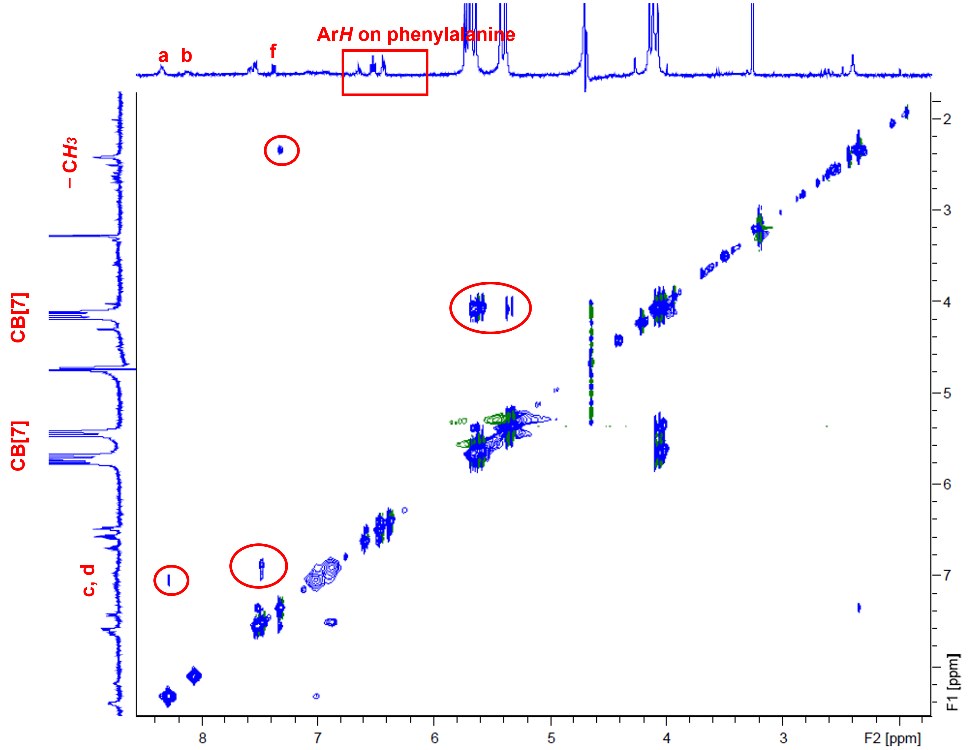


**Figure S26**. 2D NOESY NMR spectrum (400 MHz, D_2_O, 298 K) of *L*-FPy⊂CB[7] ([*L*-FPy] = 5.0×10^-4^ M, [CB[7]] = 1.0×10^-3^ M).


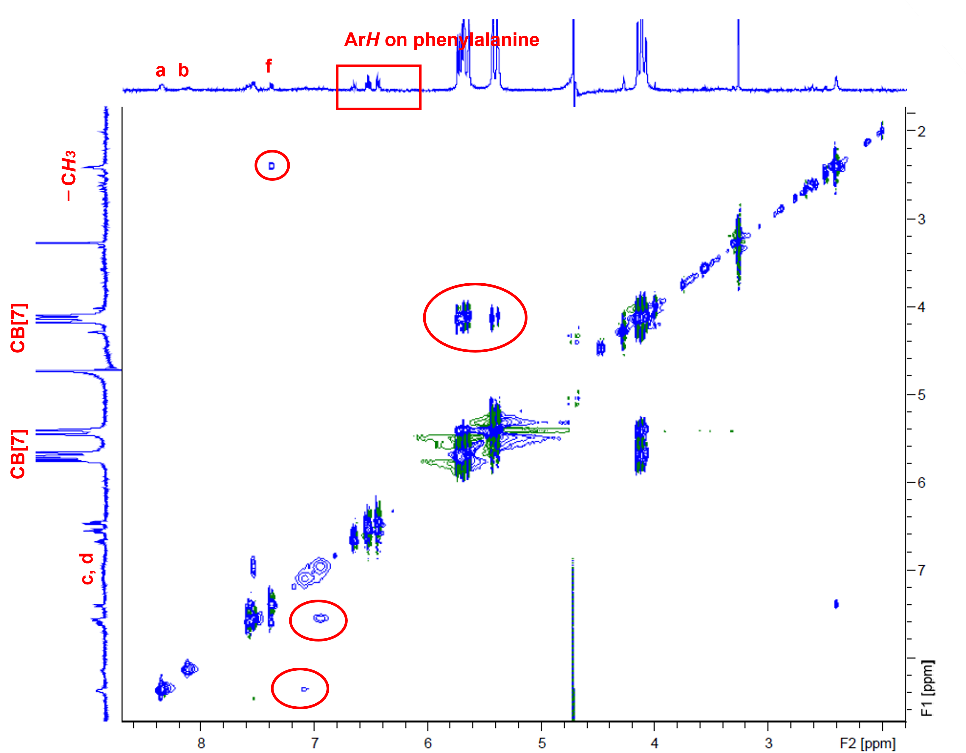


**Figure S27**. 2D NOESY NMR spectrum (400 MHz, D_2_O, 298 K) of *D*-FPy⊂CB[7] ([*D*-FPy] = 5.0×10^-4^ M, [CB[7]] = 1.0×10^-3^ M).


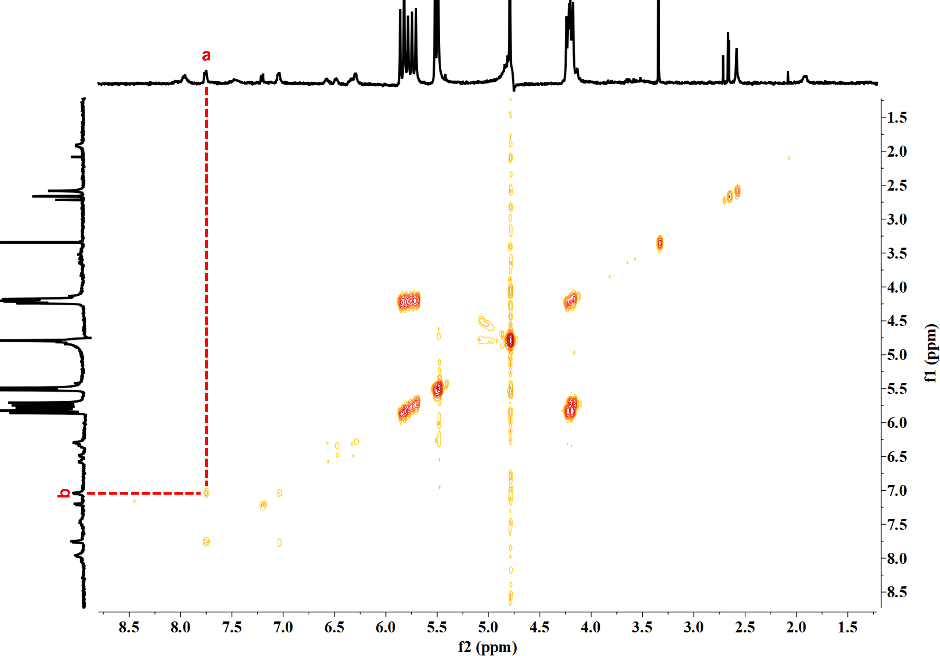


**Figure S28**. 2D ^1^H-^1^H COSY NMR spectrum (400 MHz, D_2_O, 298 K) of *L*-FPy⊂CB[8] ([*L*-FPy] = 5.0×10^-4^ M, [CB[8]] = 5.0×10^-4^ M).


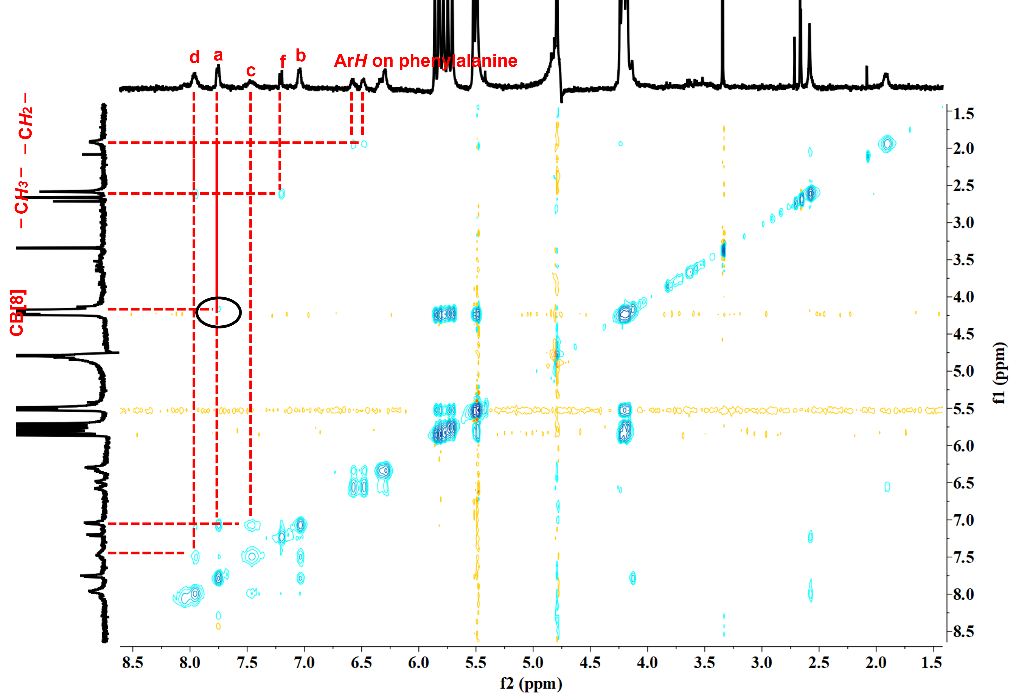


**Figure S29**. 2D NOESY NMR spectrum (400 MHz, D_2_O, 298 K) of *L*-FPy⊂CB[8] ([*L*-FPy] = 5.0×10^-4^ M, [CB[8]] = 5.0×10^-4^ M).


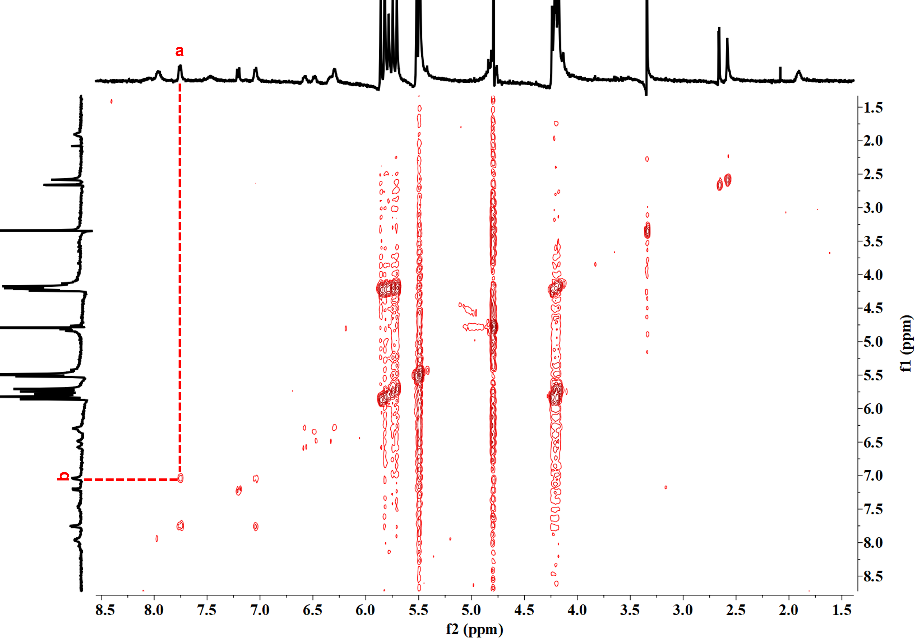


**Figure S30**. 2D ^1^H-^1^H COSY NMR spectrum (400 MHz, D_2_O, 298 K) of *D*-FPy⊂CB[8] ([*D*-FPy] = 5.0×10^-4^ M, [CB[8]] = 5.0×10^-4^ M).


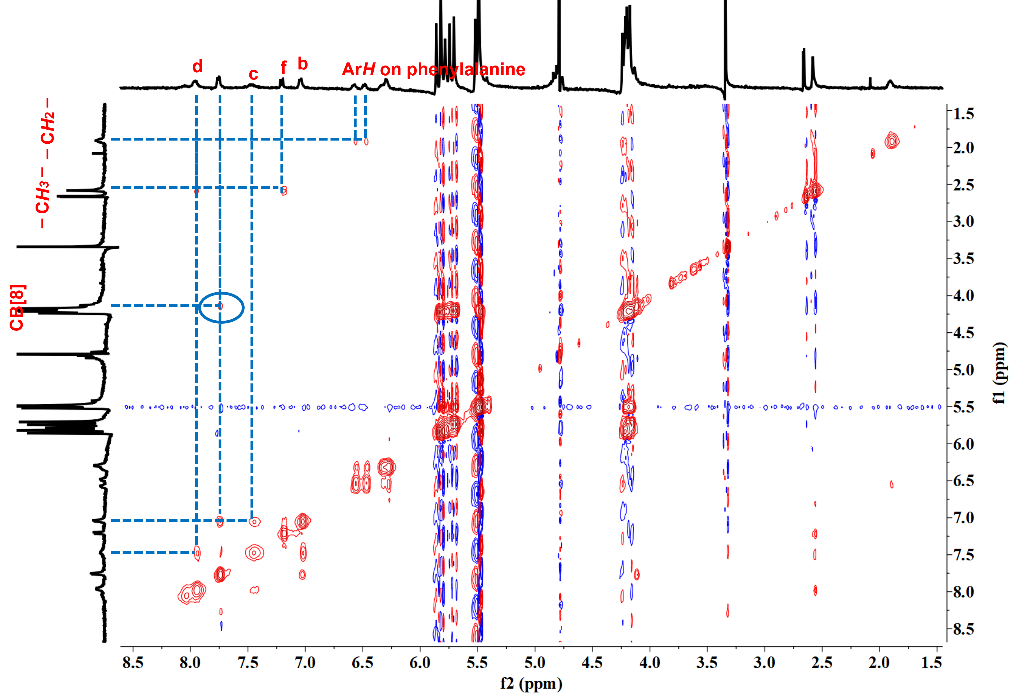


**Figure S31**. 2D NOESY NMR spectrum (400 MHz, D_2_O, 298 K) of *D*-FPy⊂CB[8] ([*D*-FPy] = 5.0×10^-4^ M, [CB[8]] = 5.0×10^-4^ M).


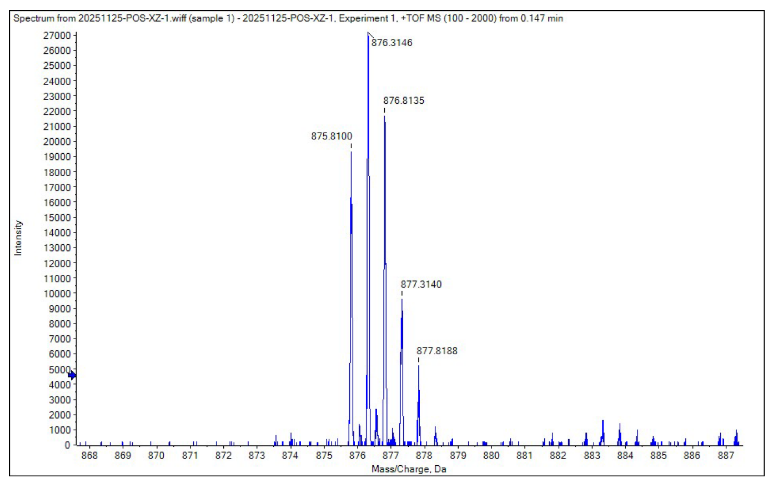


**Figure S32**. HR-MS spectra of *L*-FPy⸦CB[8].


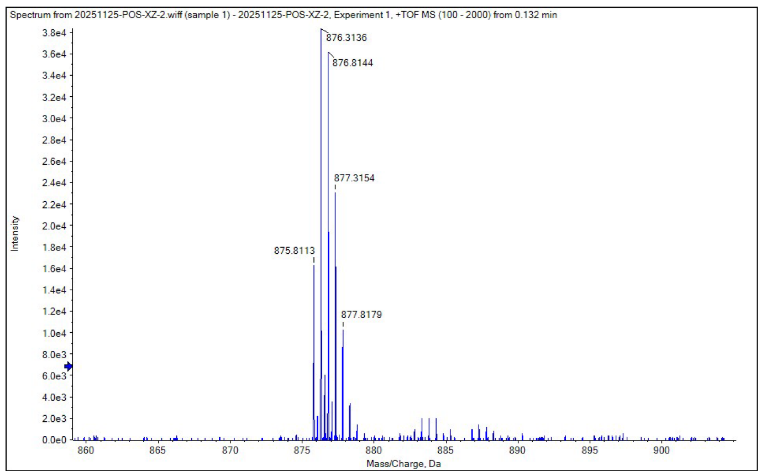


**Figure S33**. HR-MS spectra of *D*-FPy⸦CB[8].

# Characterization and Photophysical Properties of Assemblies.


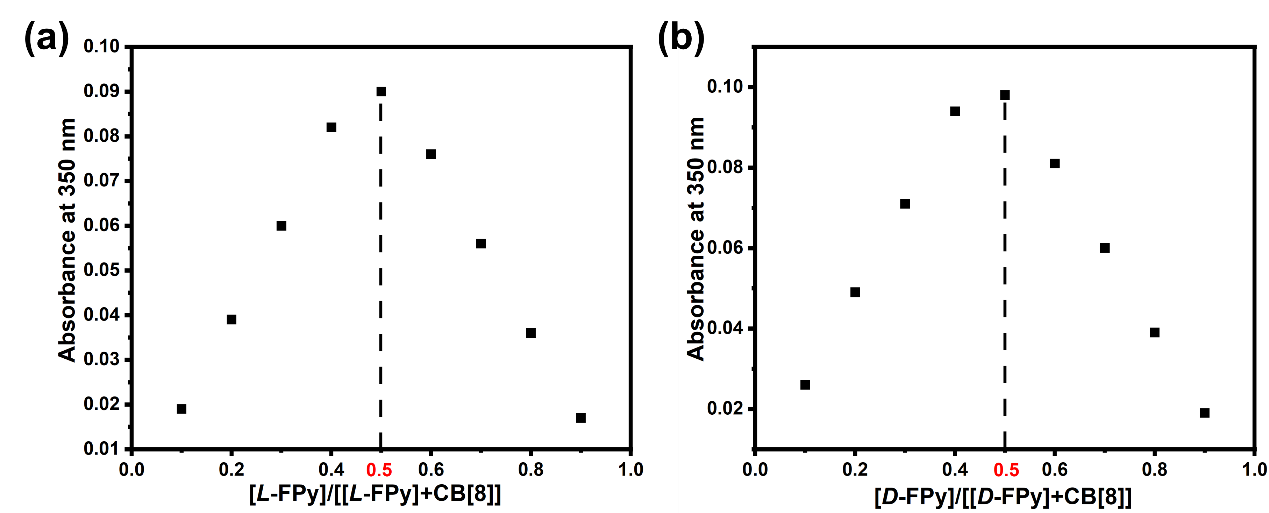


**Figure S34**. Job’s plot of (a), (b) *L*/*D*-FPy and CB[8] acquired by recording the absorbance at 350 nm at 298 K ([*L*/*D*-FPy] + [CB[8]] = 2.0×10^−5^ M).


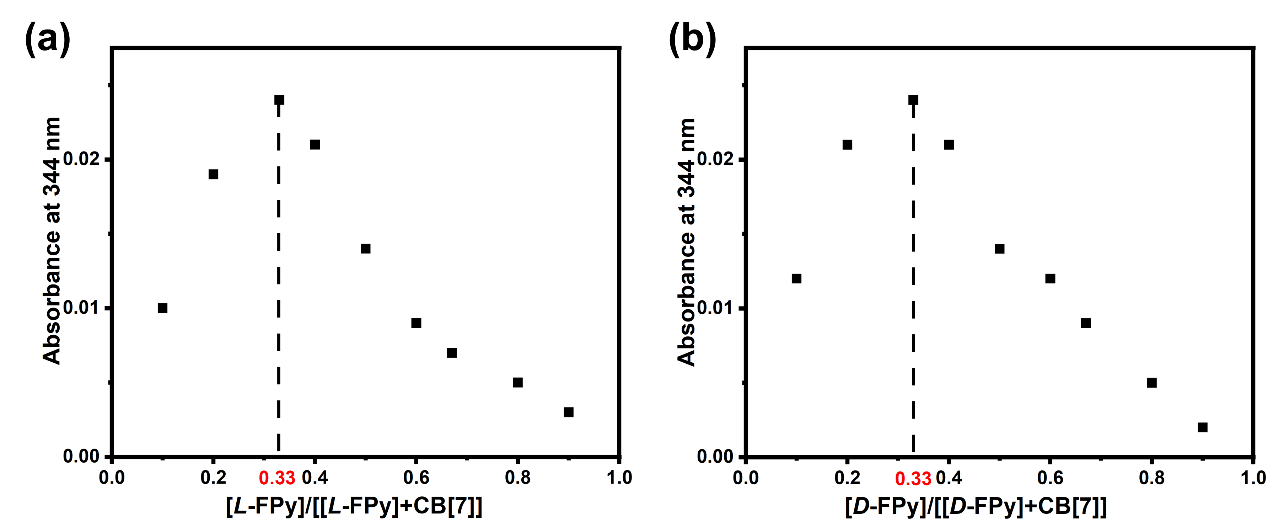


**Figure S35**. Job’s plot of (a) *L*-FPy, (b) *L*/*D*-FPy and CB[7] acquired by recording the absorbance at 344 nm at 298 K ([*L*/*D*-FPy] + [CB[7]] = 2.0×10^−5^ M).


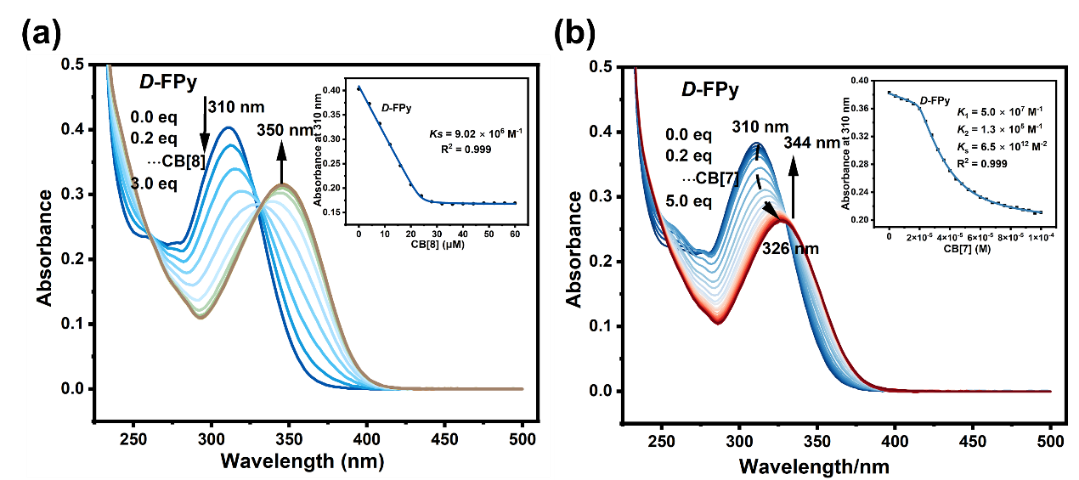


**Figure S36**. (a) UV-vis absorption spectra and absorbance intensity changes of *D*-FPy at 310 nm (inset) upon addition of CB[8] in H_2_O at 298 K. (b) UV-vis absorption spectra and absorbance intensity changes of *D*-FPy at 310 nm (inset) upon addition of CB[7] in H_2_O at 298 K. ([*D*-FPy] = 2.0×10^−5^ M, [CB[7]] = 0－1.0×10^−4^ M and [CB[8]] = 0－6.0×10^−5^ M)


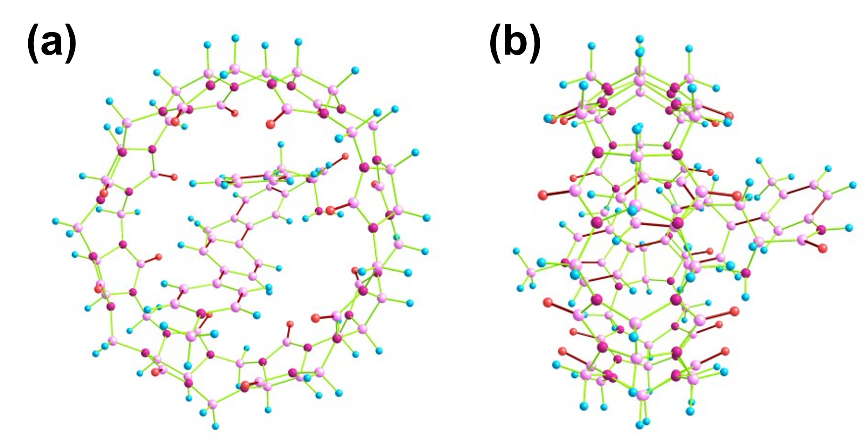


**Figure S37**. (a) (b) Optimized structure of *D*-FPy⊂CB[8].


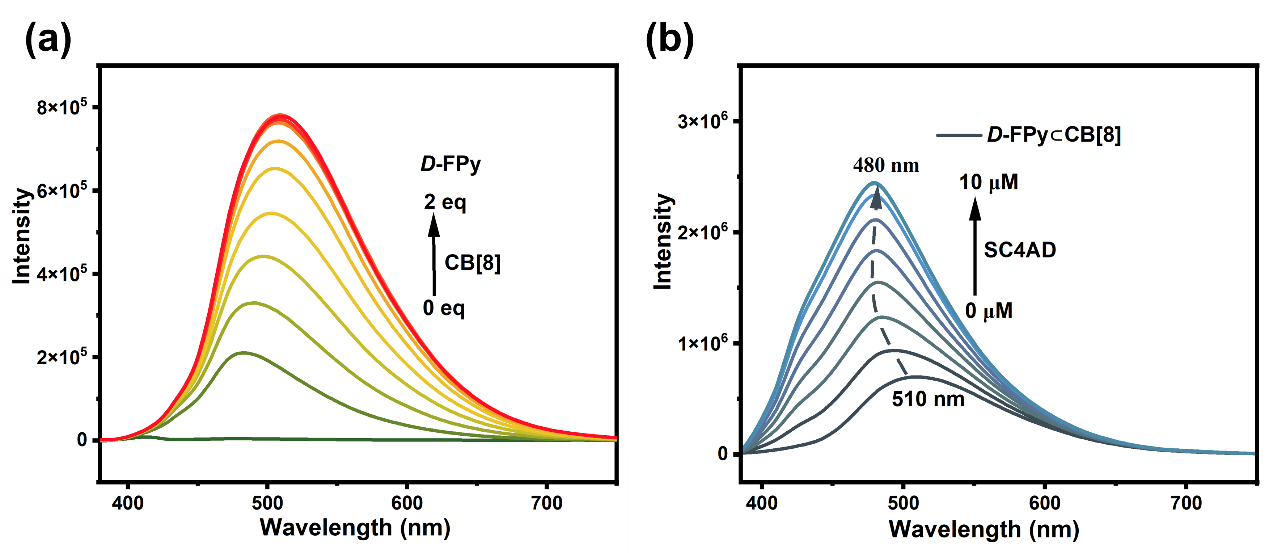


**Figure S38**. (a) Fluorescence emission spectral changes of *D*-FPy upon addition of 0, 0.2, 0.4, 0.6, 0.8, 1.0, 1.2, 1.4, 1.6, 1.8, and 2.0 equivalent CB[8] in H_2_O at 298 K. ([*D*-FPy] = 2.0×10^−5^ M, 𝜆_ex_ = 350 nm). (b) Fluorescence emission spectral of *D*-FPy⊂CB[8] ([*D*-FPy] = [CB[8]] = 2.0×10^−5^ M) upon the addition of 0－10×10^−6^ M SC4AD in aqueous solution.


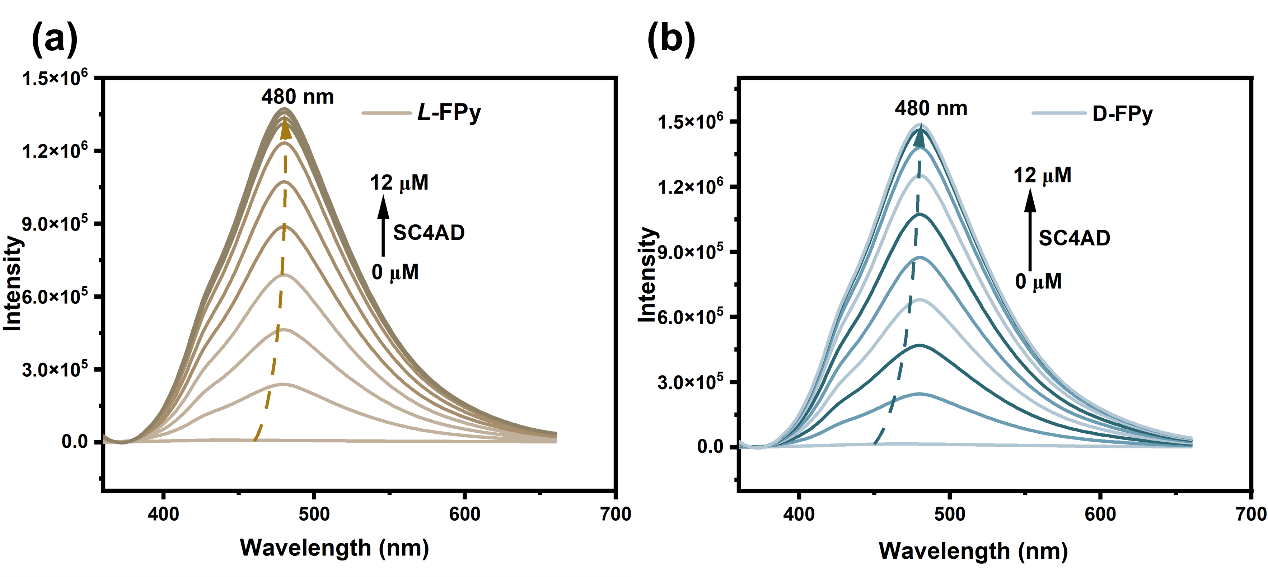


**Figure S39**. Fluorescence emission spectral of (a) *L*-FPy, (b) *D*-FPy upon the addition of 0－12×10^−6^ M SC4AD in aqueous solution.


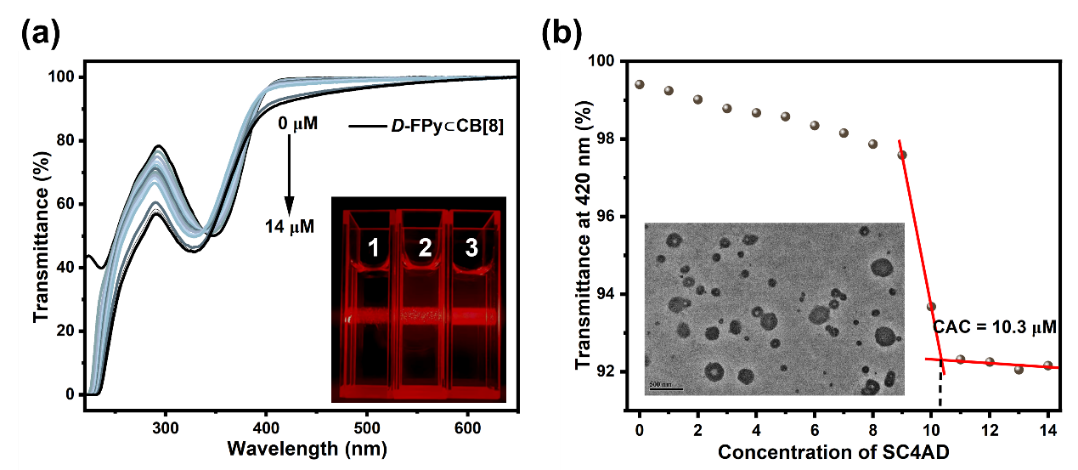


**Figure S40**. (a) Transmittance changes of *D*-FPy⊂CB[8] in varying concentration of SC4AD from 0 to 14×10^−6^ M. Inset: Tyndall effect of *D*-FPy (1), *D*-FPy⊂CB[8] (2), and *D*-FPy⊂CB[8]@SC4AD (3). (b) Dependence of the optical transmittance at 420 nm upon addition of SC4AD. Inset: Transmission electron microscopy image of *D*-FPy⊂CB[8]@SC4AD assembly. ([*D*-FPy] = [CB[8]] = 2.0×10^−5^ M, [SC4AD] = 10×10^−6^ M).

**
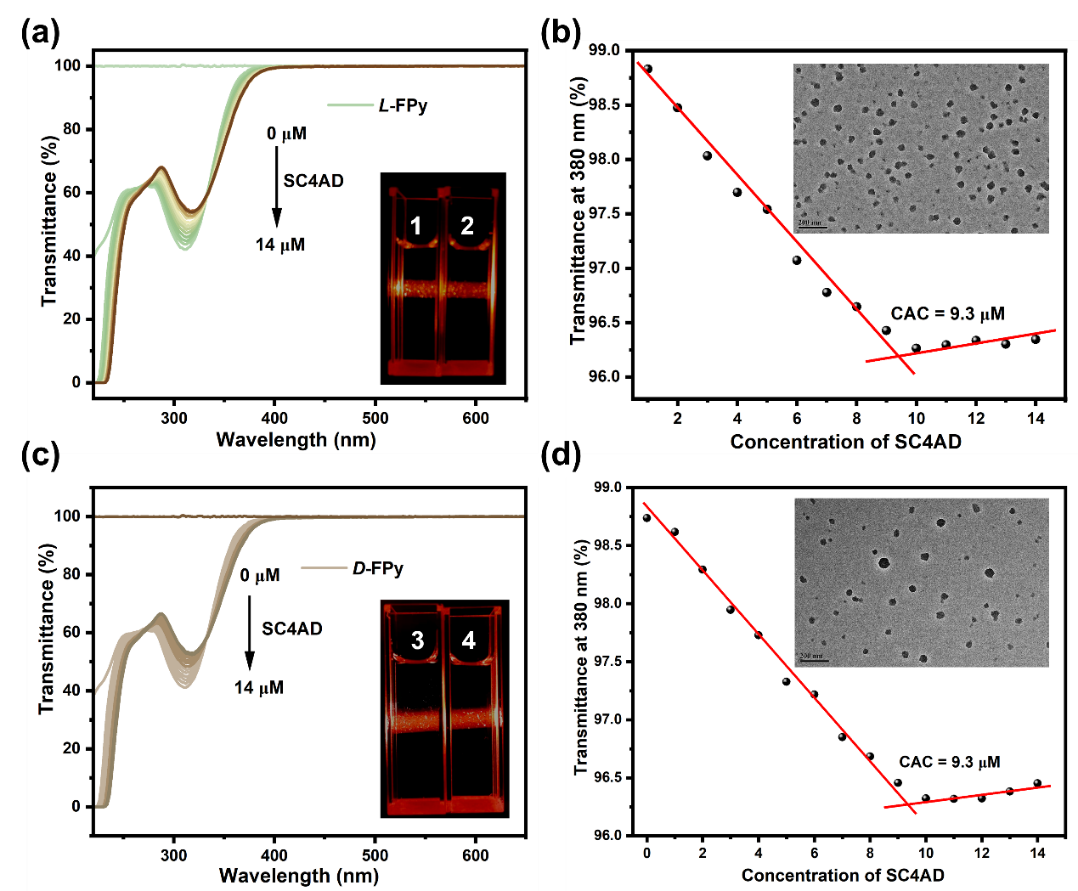
**

**Figure S 41**. (a) Transmittance changes of *L*-FPy in varying concentrations of SC4AD from 0 to 14×10^−6^ M. Inset: Tyndall effect of *L*-FPy (1) and *L*-FPy@SC4AD (2). (b) Dependence of the optical transmittance at 380 nm upon addition of SC4AD. Inset: Transmission electron microscopy image of *L*-FPy@SC4AD assembly. (c) Transmittance changes of *D*-FPy in varying concentration of SC4AD from 0 to 14×10^−6^ M. Inset: Tyndall effect of *D*-FPy (3) and *D*-FPy@SC4AD (4). (d) Dependence of the optical transmittance at 380 nm upon addition of SC4AD. Inset: Transmission electron microscopy image of *L*-FPy@SC4AD assembly. ([*L*-FPy] = [*D*-FPy] = 2.0×10^−5^ M, [SC4AD] = 14×10^−6^ M)


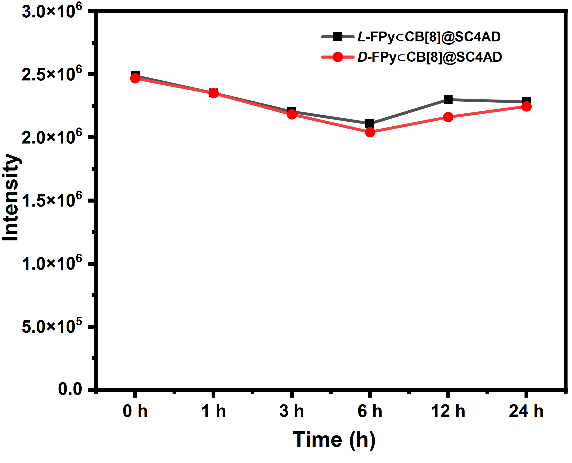


**Figure S 42**. The fluorescence intensity changes of *L*/*D*-FPy⊂CB[8]@SC4AD assembly at room temperature within 24 hours. ([*L*-FPy] = [*D*-FPy] = [CB[8]] = 2.0×10^−5^ M, [SC4AD] = 10×10^−6^ M).


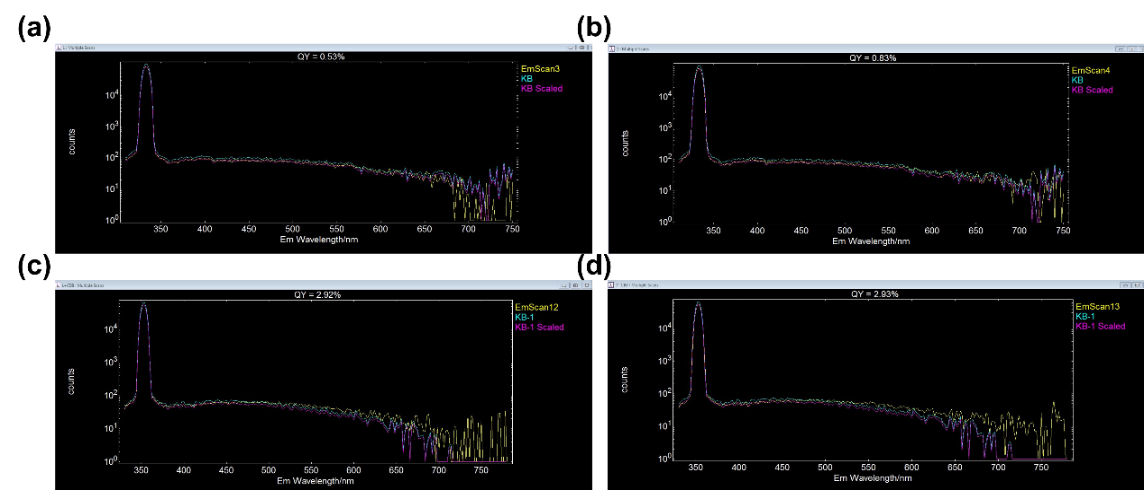


**Figure S43**. The QY of (a) *L*-FPy, (b) *D*-FPy, (c) *L*-FPy⊂CB[8], and (d) *D*-FPy⊂CB[8].


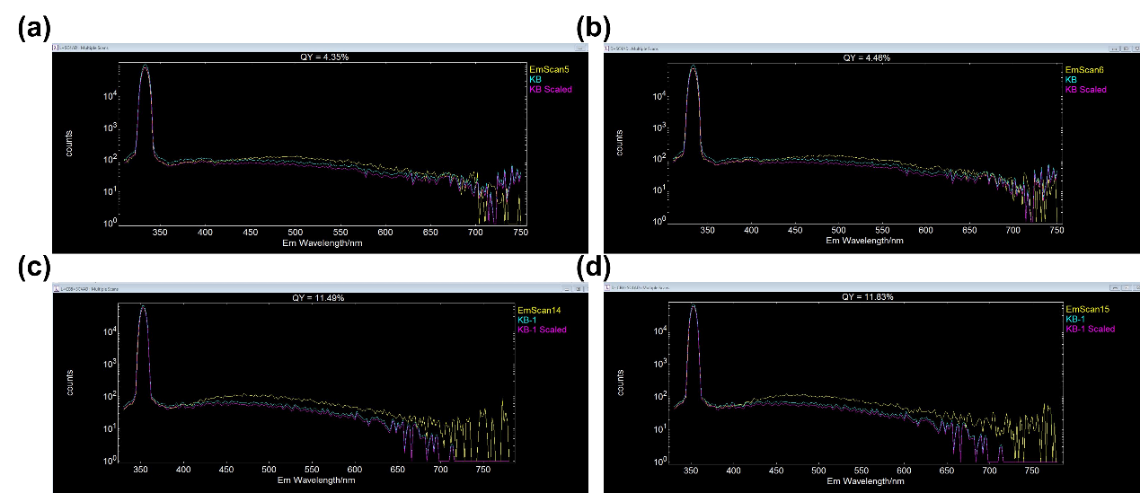


**Figure S44**. The QY of (a) *L*-FPy@SC4AD, (b) *D*-FPy@SC4AD, (c) *L*-FPy⊂CB[8]@SC4AD and (d) *D*-FPy⊂CB[8]@SC4AD.


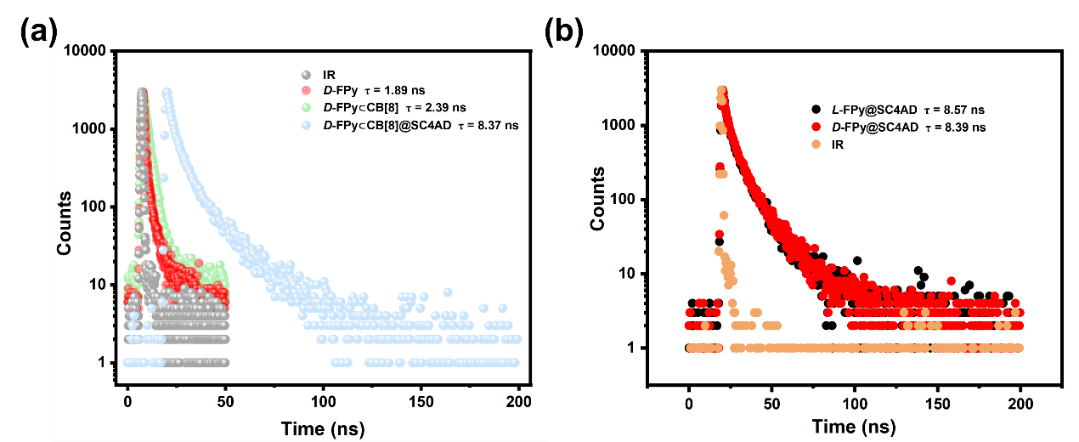


**Figure S45**. (a) Time-resolved photoluminescence decay curves of *D*-FPy, *D*-FPy⊂CB[8] and *D*-FPy⊂CB[8]@SC4AD in aqueous solution at 298 K. (b) Time-resolved photoluminescence decay curves of *L*/*D*-FPy@SC4AD at 480 nm in aqueous solution at 298 K.

**3.1 Energy-transfer efficiency (Φ_ET_)**

Energy-transfer efficiency, **Φ_ET_**, the fraction of the absorbed energy that is transferred to the acceptor is experimentally measured as a ratio of the fluorescence intensities of the donor in the absence and presence of the acceptor (I_D_ and I_DA_).

$$\boldsymbol{\Phi}_{\mathbf{ET}}\boldsymbol{=1-}\frac{\mathbf{I}_{\mathbf{DA}}}{\mathbf{I}_{\mathbf{D}}}$$

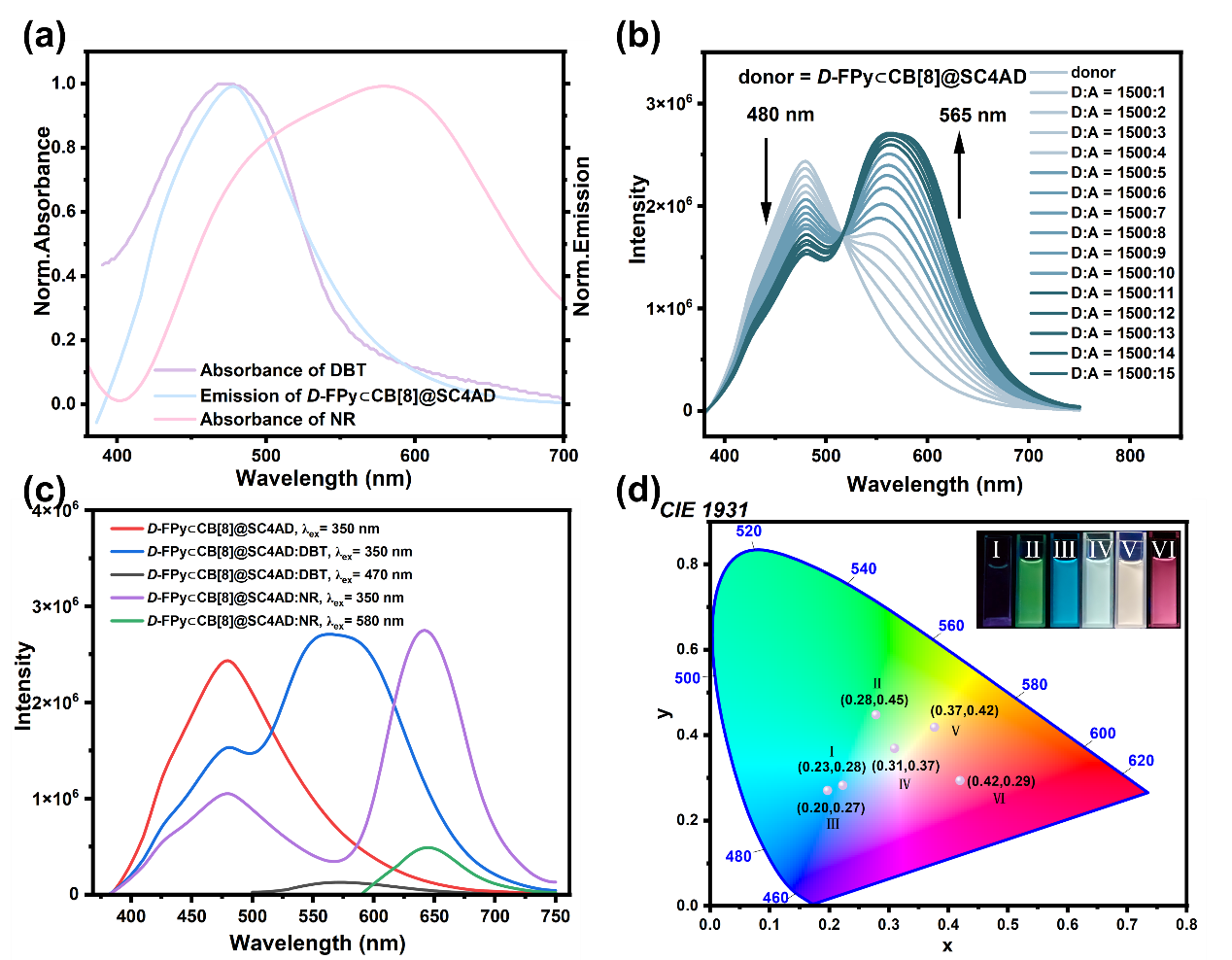


**Figure S46**. (a) Normalized emission spectrum of *D*-FPy⊂CB[8]@SC4AD assembly and absorption spectra of DBT, NR. (b) Fluorescence emission spectrum of *D*-FPy⊂CB[8]@SC4AD:DBT at different donor/acceptor ratios in aqueous solution at 298 K. (c) Fluorescence emission spectra of *D*-FPy⊂CB[8]@SC4AD (λ_ex_ = 350 nm), *D*-FPy⊂CB[8]@SC4AD:DBT (λ_ex_ = 350 nm), *D*-FPy⊂CB[8]@SC4AD:DBT (λ_ex_ = 470 nm), *D*-FPy⊂CB[8]@SC4AD:NR (λ_ex_ = 350 nm) and *D*-FPy⊂CB[8]@SC4AD:NR (λ_ex_ = 580 nm) in aqueous solution at 298 K. (d) CIE 1931 chromaticity diagram and inset: Photographs of the aqueous solutions of *D*-FPy (Ⅰ), *D*-FPy⊂CB[8] (Ⅱ), *D*-FPy⊂CB[8]@SC4AD (Ⅲ), *D*-FPy⊂CB[8]@SC4AD:DBT (Ⅳ, Ⅴ), *D-*FPy⊂CB[8]@SC4AD:NR (Ⅵ). ([*L*-FPy] = [CB[8]] = 2.0×10^−5^ M, [SC4AD] = 10×10^−6^ M, [DBT] = 2.0×10^−7^ M, [NR] = 3.33×10^−7^ M, λ_ex_ = 350 nm, 298 K).

**3.2 Antenna efficiency (AE)**

The antenna effect under certain concentrations of donor and acceptor equals the ratio of the emission of the acceptor upon excitation of the donor.

$$\mathbf{Antenna} \mathbf{effect}\mathbf{=}\frac{\mathbf{I}_{\mathbf{A+D}\left( \boldsymbol{\lambda}_{\mathbf{ex}}\boldsymbol{=}\mathbf{donor} \right)}\boldsymbol{-}\mathbf{I}_{\mathbf{D}\left( \boldsymbol{\lambda}_{\mathbf{ex}}\boldsymbol{=}\mathbf{donor} \right)}}{\mathbf{I}_{\mathbf{A+D}\left( \boldsymbol{\lambda}_{\mathbf{ex}}\boldsymbol{=}\mathbf{acceptor} \right)}}$$

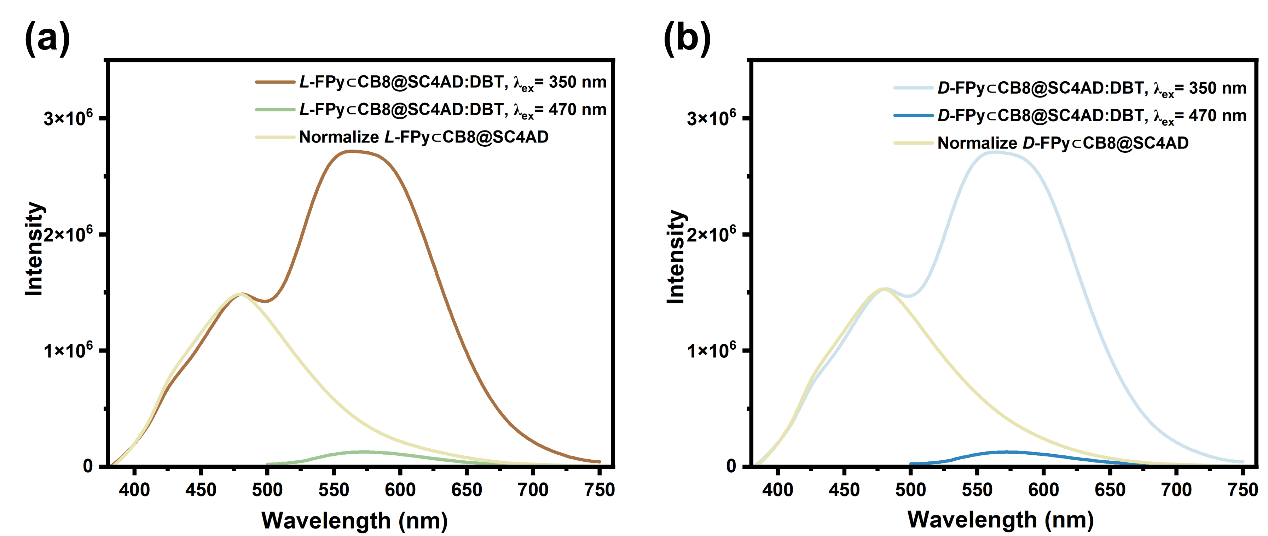


**Figure S47**. Antenna effect maxima of (a) *L*-FPy⸦CB[8]@SC4AD:DBT, (b) *D*-FPy⸦CB[8]@SC4AD:DBT.


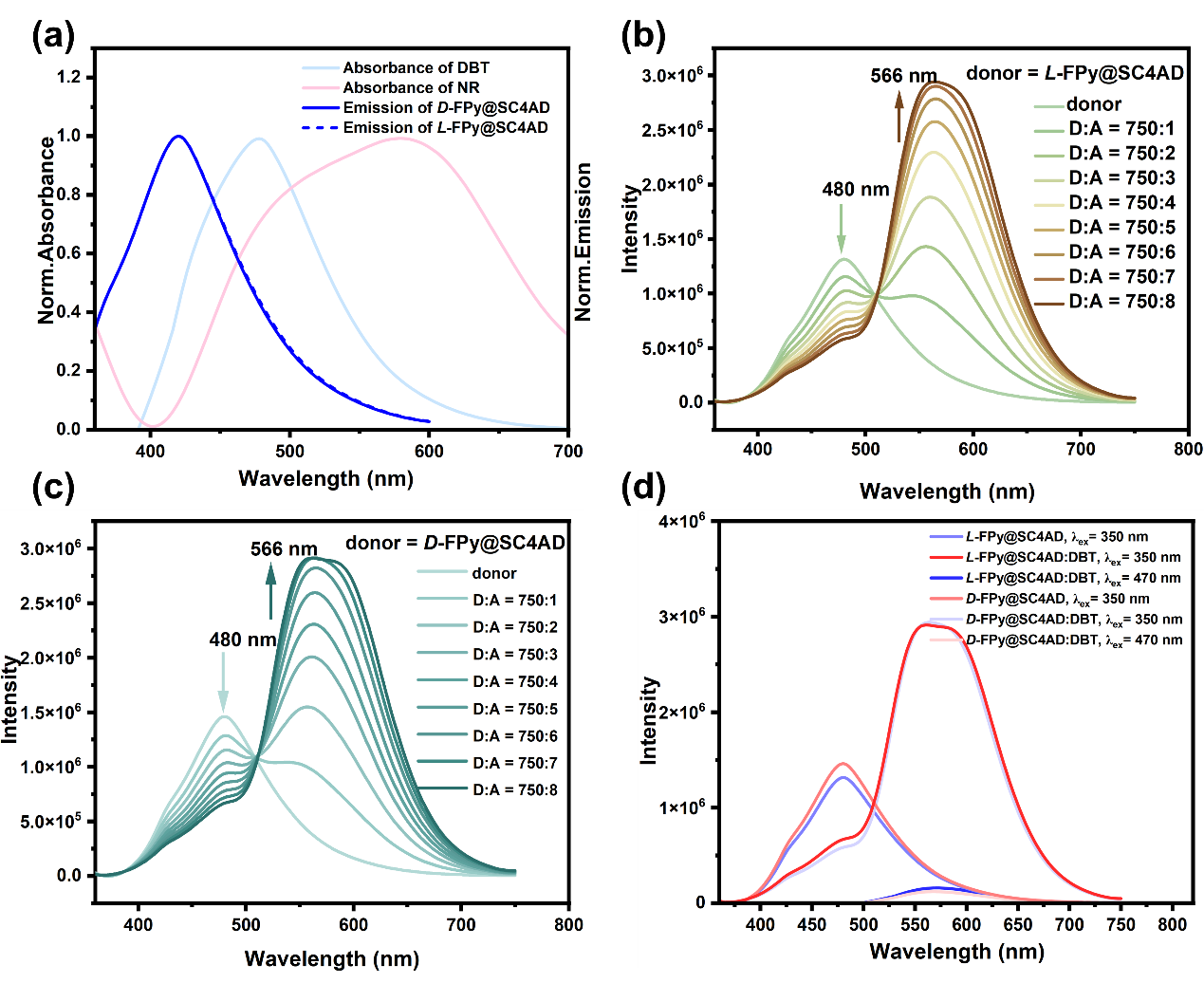


**Figure S48**. (a) Normalized emission spectrum of *L*/*D*-FPy@SC4AD assembly and absorption spectra of DBT, NR. (b) (c) Fluorescence emission spectrum of *L*/*D*-FPy@SC4AD:DBT at different donor/acceptor ratios in aqueous solution at 298 K. (d) Fluorescence emission spectra of *L*/*D*-FPy@SC4AD (λ_ex_ = 350 nm), *L*/*D*-FPy@SC4AD:DBT (λ_ex_ = 350 nm), *L*/*D*-FPy@SC4AD:DBT (λ_ex_ = 470 nm) in aqueous solution at 298 K.


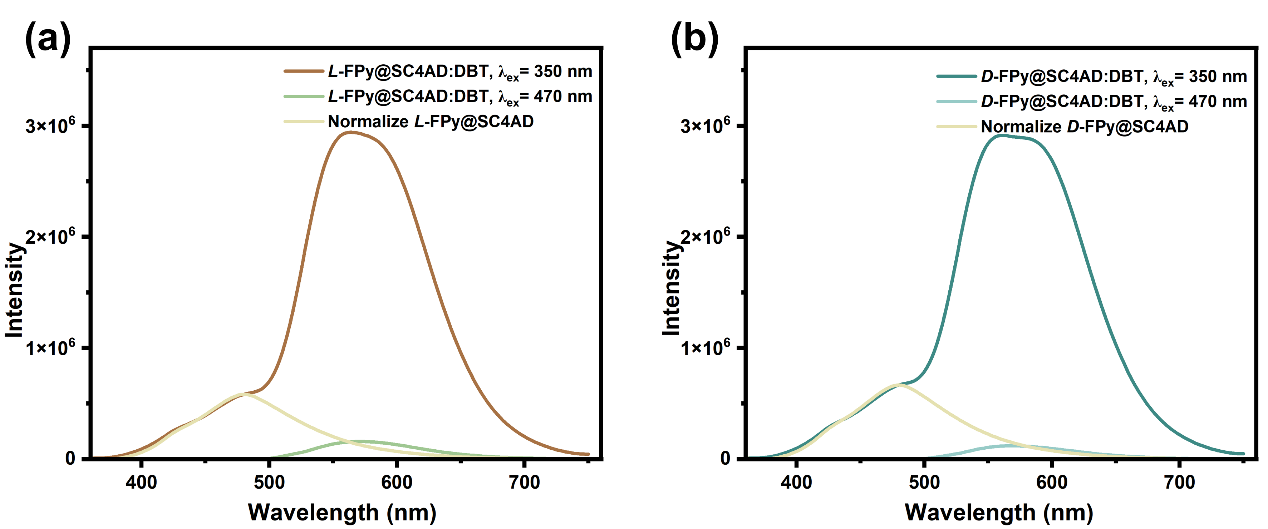


**Figure S49**. Antenna effect maxima of (a) *L*-FPy@SC4AD:DBT, (b) *D*-FPy@SC4AD:DBT.


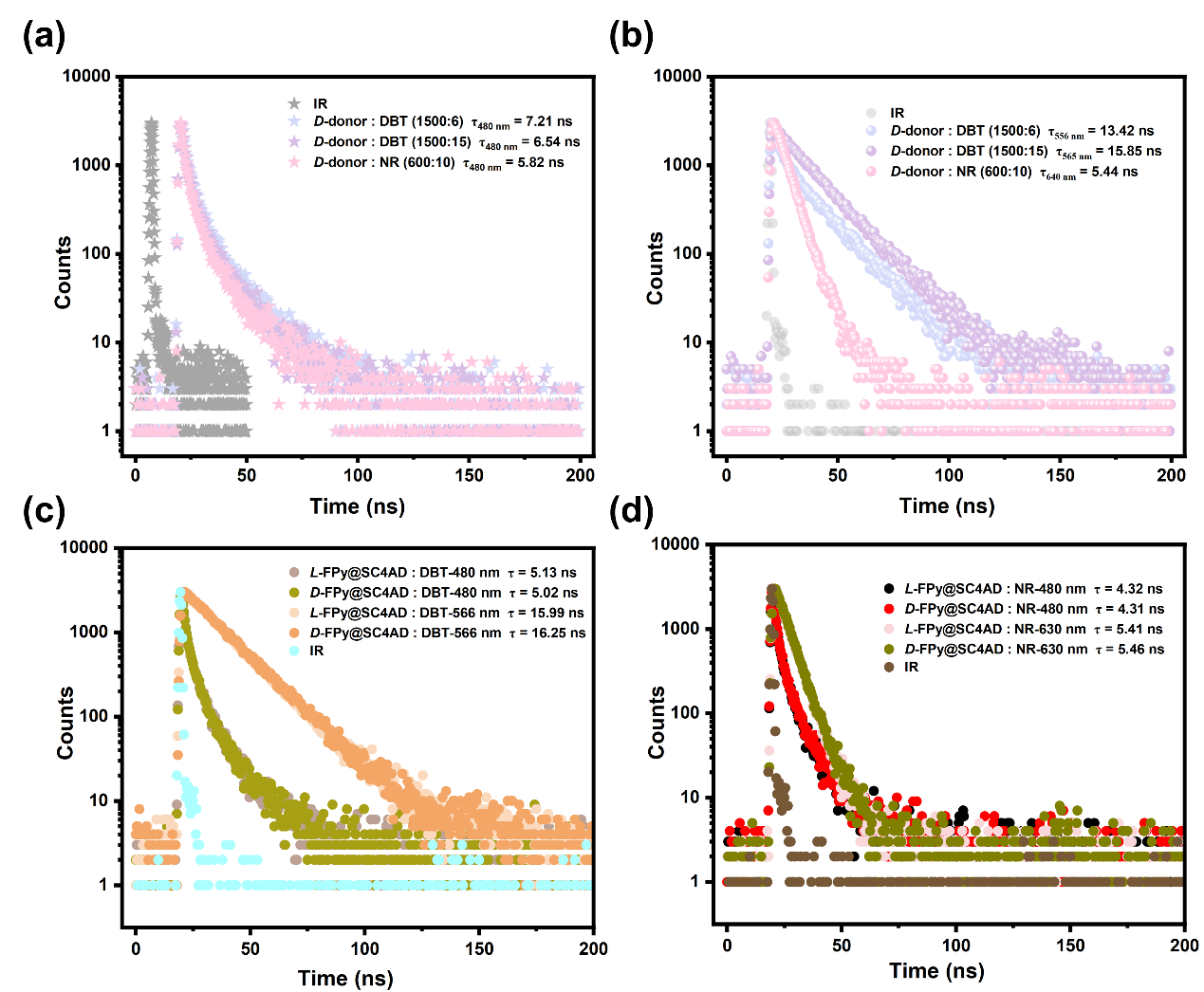


**Figure S50**. (a) (b) Time-resolved photoluminescence decay curves of *D*-FPy⊂CB[8]@SC4AD:DBT and *D*-FPy⊂CB[8]@SC4AD:NR in aqueous solution at 298 K. (c) (d) Time-resolved photoluminescence decay curves of *L*/*D*-FPy@SC4AD:DBT and *L*/*D*-FPy@SC4AD:NR in aqueous solution at 298 K.


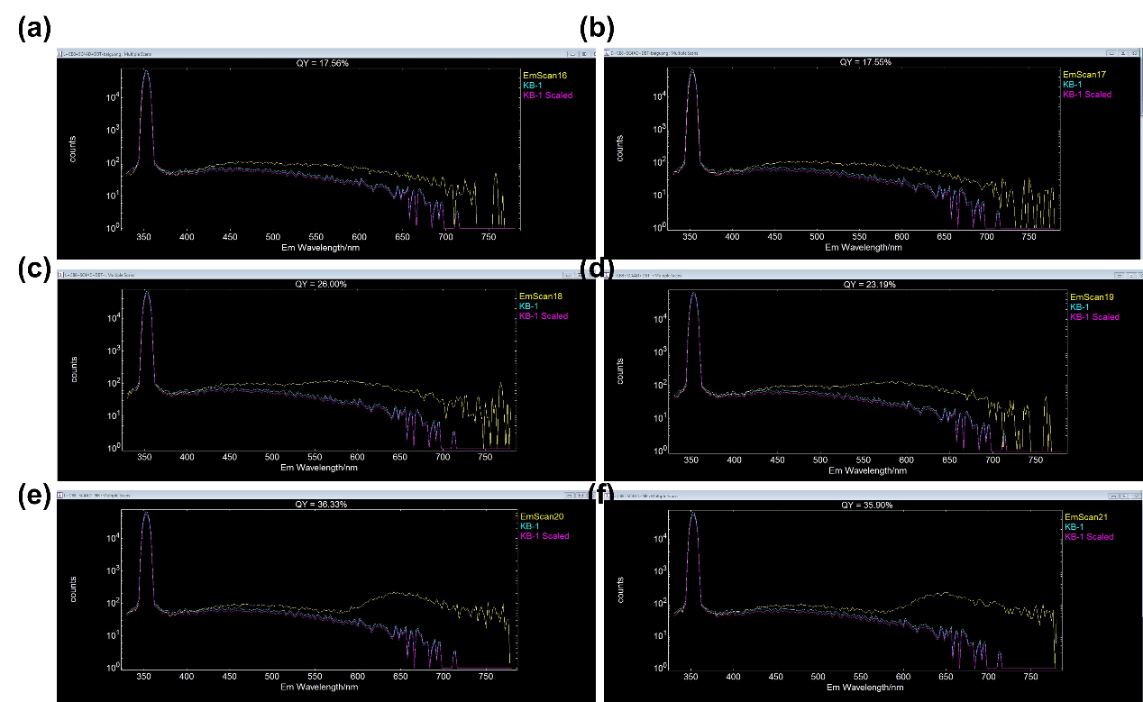


**Figure S51.** The QY of (a) *L*-FPy⊂CB[8]@SC4AD:DBT (1500:6), (b) *D*-FPy⊂CB[8]@SC4AD:DBT (1500:6), (c) *L*-FPy⊂CB[8]@SC4AD:DBT (1500:15), (d) *D*-FPy⊂CB[8]@SC4AD:DBT (1500:15), (e) *L*-FPy⊂CB[8]@SC4AD:NR (600:10) (f) *D*-FPy⊂CB[8]@SC4AD:NR (600:10).


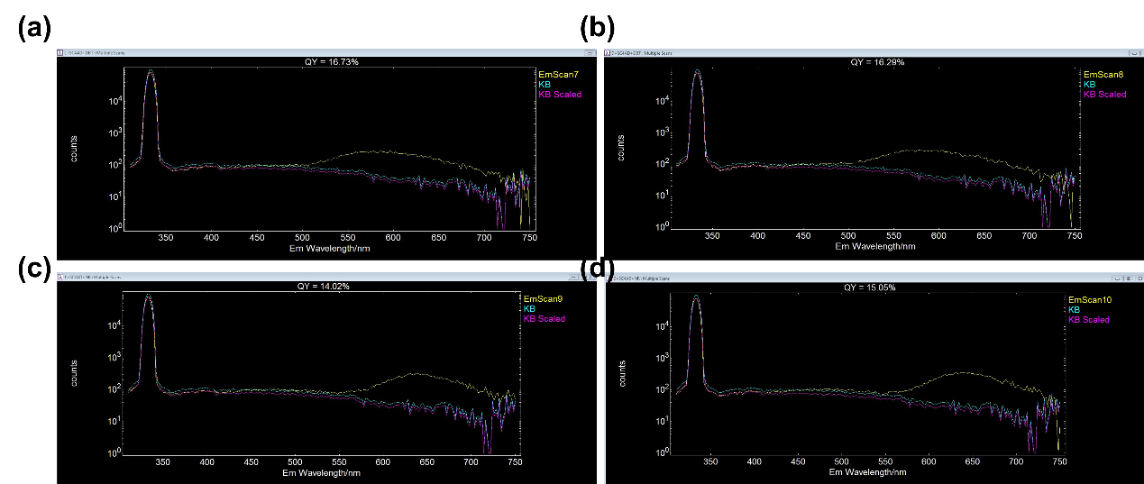


**Figure S52**. The QY of (a) *L*-FPy@SC4AD:DBT (1500:15), (b) *D*-FPy@SC4AD:DBT (1500:15), (c) *L* -FPy@SC4AD:NR(600:10), (d) *D*-FPy@SC4AD:NR(600:10).

*
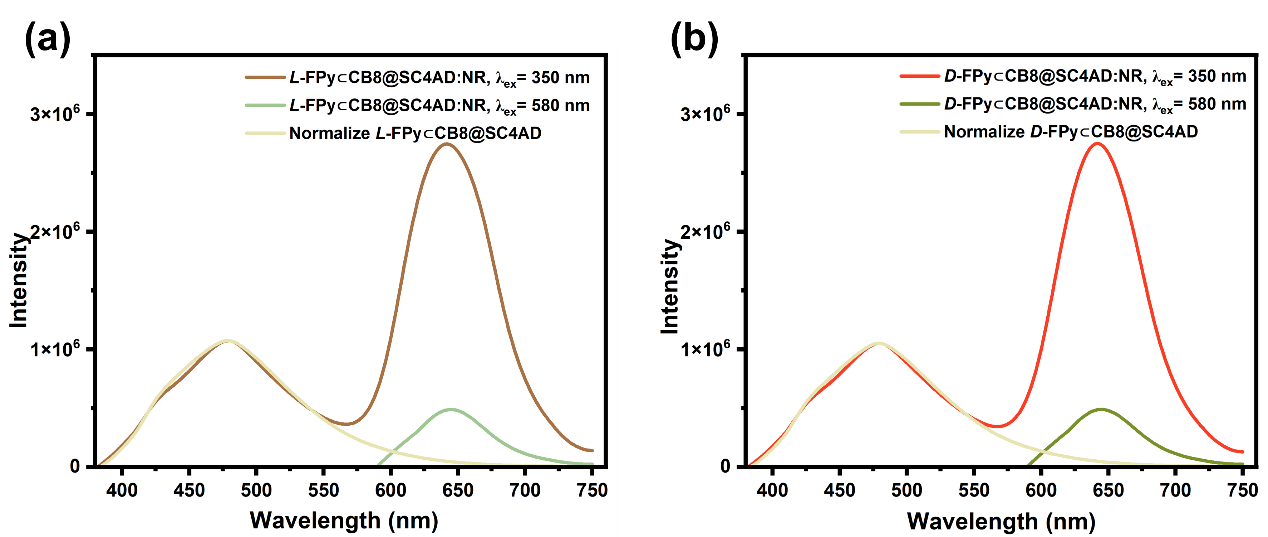
*

**Figure S53**. Antenna effect maxima of (a) *L*-FPy⊂CB[8]@SC4AD:NR, (b) *D*-FPy⊂CB[8]@SC4AD:NR.


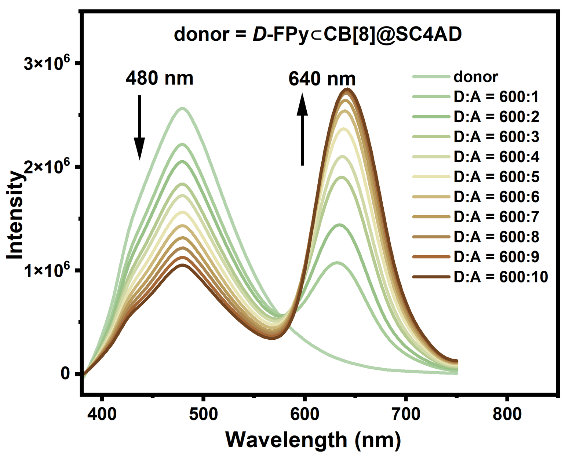


**Figure S54**. Fluorescence emission spectrum of *D*-FPy⊂CB[8]@SC4AD:NR at different donor/acceptor ratios in aqueous solution at 298 K.


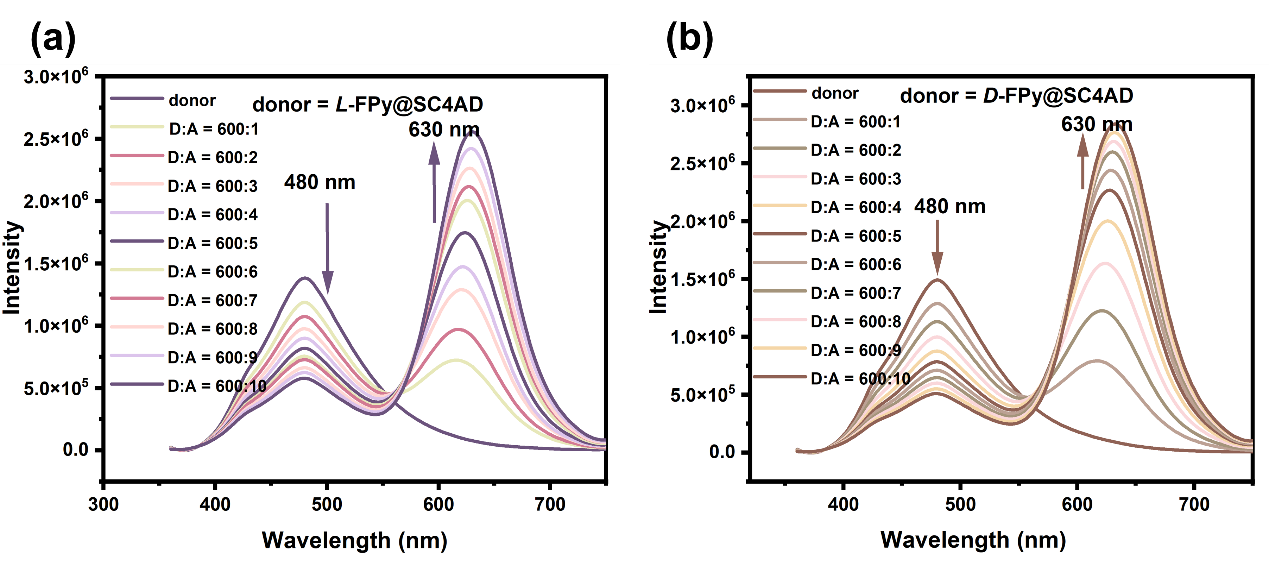


**Figure S55**. Fluorescence emission spectrum of (a) *L*-FPy@SC4AD:NR, (b) *D*-FPy@SC4AD:NR at different donor/acceptor ratios in aqueous solution at 298 K.


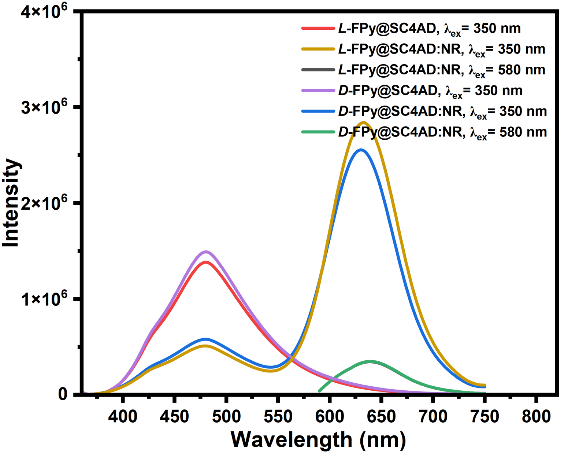


**Figure S56**. Fluorescence emission spectra of *L*/*D*-FPy@SC4AD (λ_ex_ = 350 nm), *L*/*D*-FPy@SC4AD:NR (λ_ex_ = 350 nm), *L*/*D*-FPy@SC4AD:NR (λ_ex_ = 580 nm) in aqueous solution at 298 K.


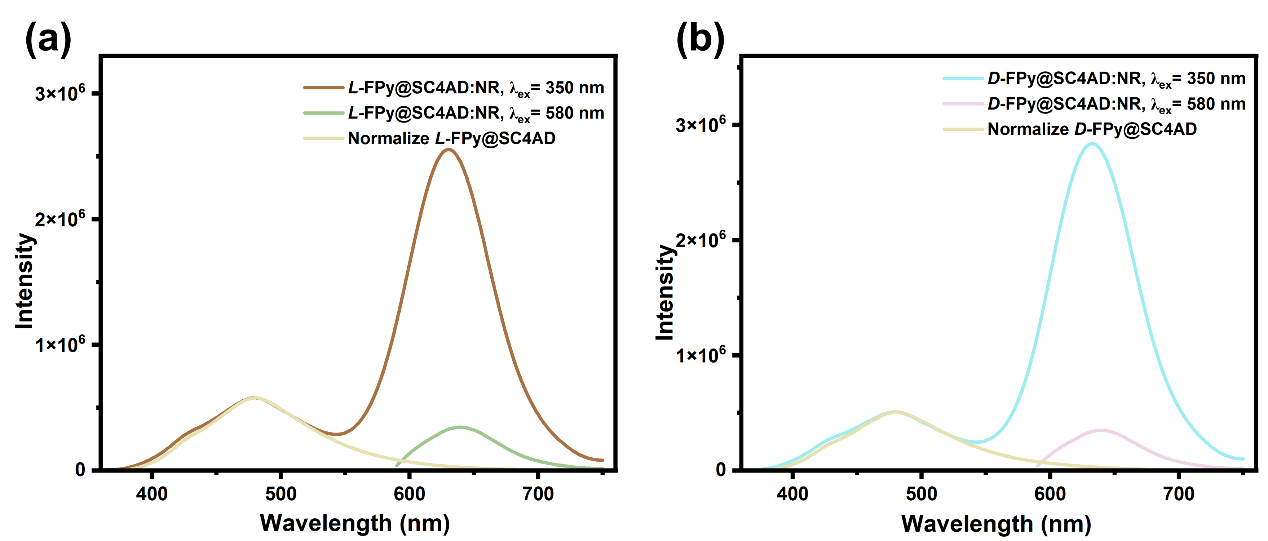


**Figure S57**. Antenna effect maxima of (a) *L*-FPy@SC4AD:NR, (b) *D*-FPy@SC4AD:NR.


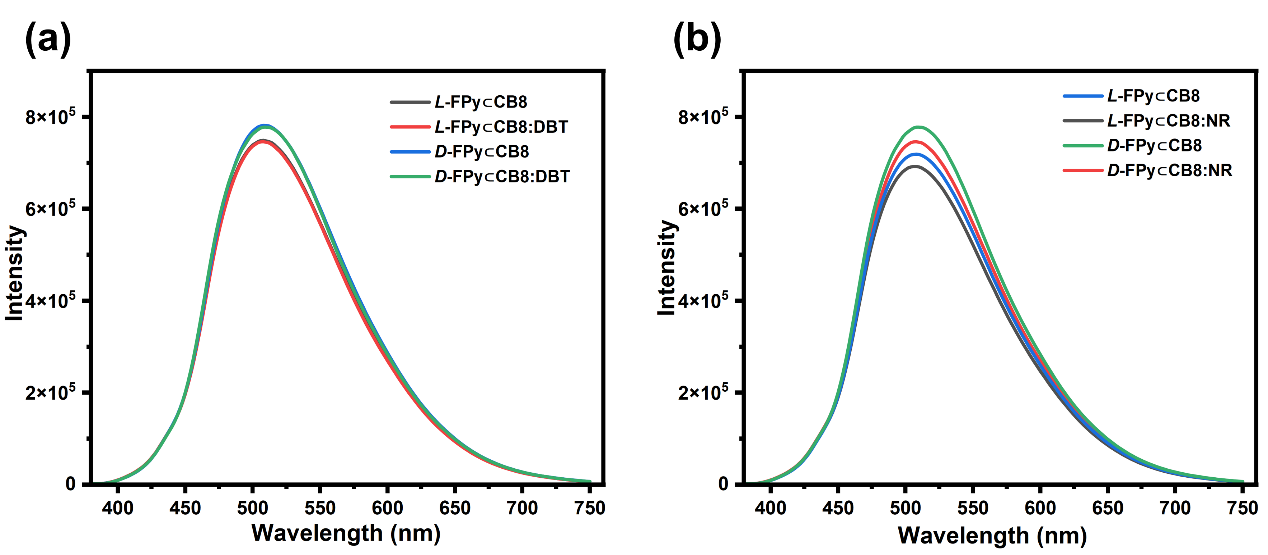


**Figure S58**. (a) Fluorescence emission spectrum of *L*/*D*-FPy⊂CB[8]:DBT in aqueous solution at 298 K. (b) Fluorescence emission spectrum of *L*/*D*-FPy⊂CB[8]:NR in aqueous solution at 298 K.

**
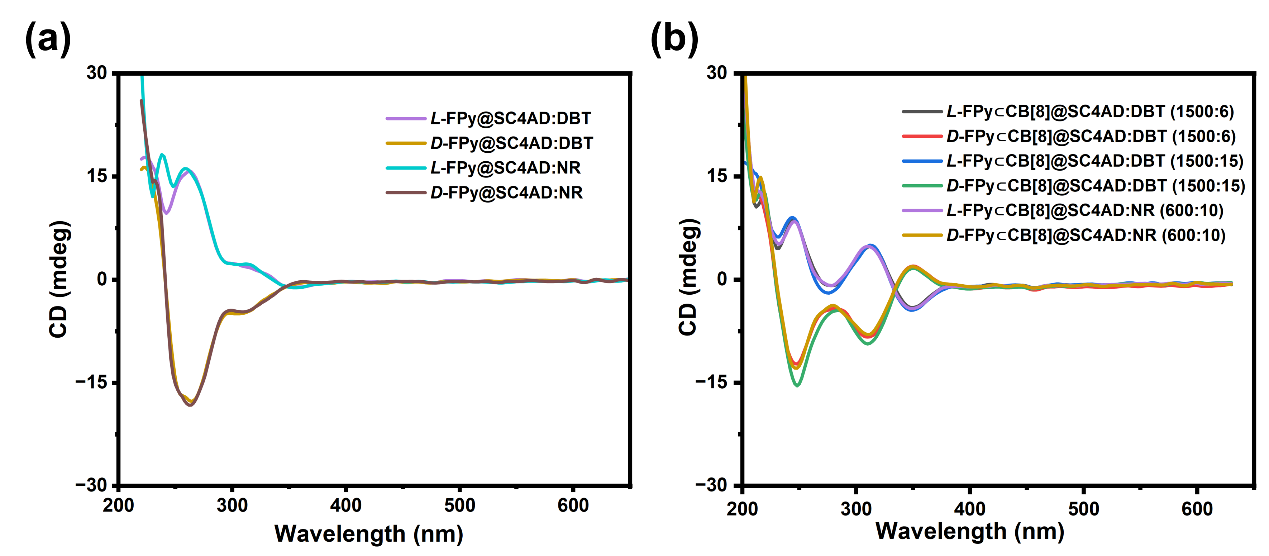
**

**Figure S59**. (a) CD spectra of *L*/*D*-FPy@SC4AD:DBT and *L*/*D*-FPy@SC4AD:NR in aqueous solution at 298 K. (b) CD spectra of *L*/*D*-FPy⊂CB[8]@SC4AD:DBT and *L*/*D*-FPy⊂CB[8]@SC4AD:NR in aqueous solution at 298 K. ([*L*-FPy] = *D*-FPy] = 1.0×10^−4^ M, [CB[8]] = 1.0×10^−4^ M and [SC4AD] = 5.0×10^−5^ M, [DBT] = 1.0×10^−6^ M, [NR] = 1.67×10^−6^ M).


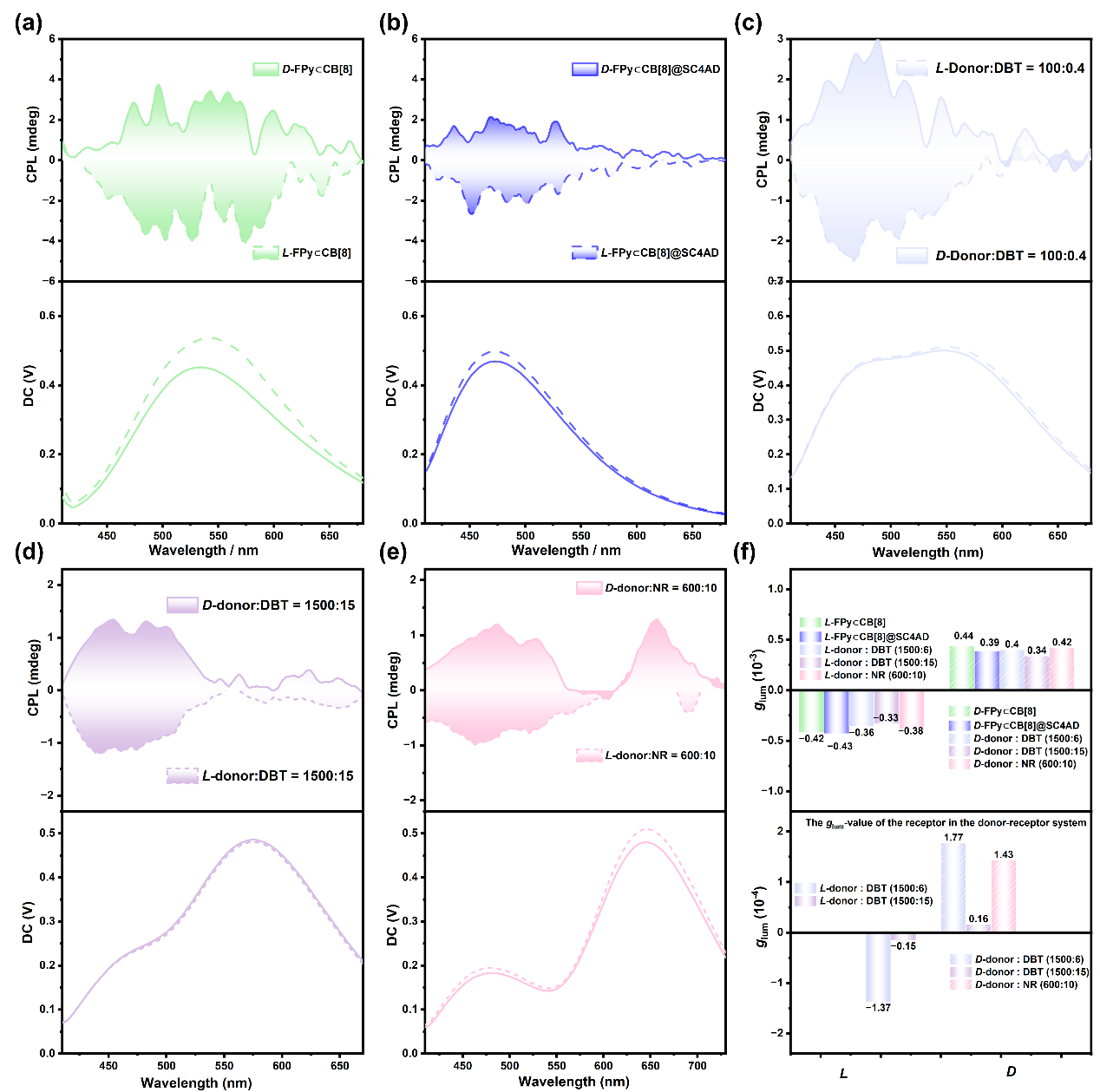


**Figure S60**. (a-e) CPL spectra of *L*/*D*-FPy⊂CB[8], *L*/*D*-FPy⊂CB[8]@SC4AD, *L*/*D*-FPy⊂CB[8]@SC4AD:DBT and *L*/*D*-FPy⊂CB[8]@SC4AD:NR in aqueous solution at 298 K. ([*L*/*D*-FPy] = *L*/*D*-FPy] = 1.0×10^−4^ M, [CB[8]] = 1.0×10^−4^ M and [SC4AD] = 5.0×10^−5^ M, [DBT] = 1.0×10^−6^ M, [NR] = 1.67×10^−6^ M).


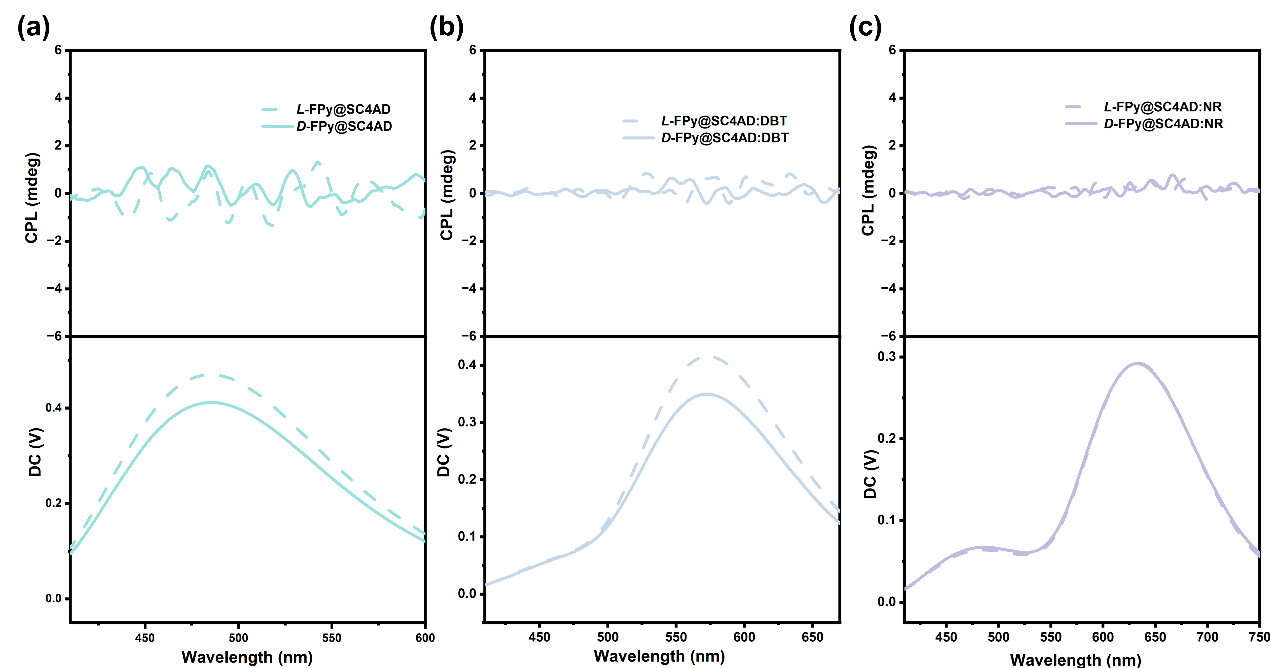


**Figure S61**. (a-c) CPL spectra of *L*/*D*-FPy@SC4AD, *L*/*D*-FPy@SC4AD:DBT and *L*/*D*-FPy@SC4AD:NR in aqueous solution at 298 K. ([*L*/*D*-FPy] = *L*/*D*-FPy] = 1.0×10^−4^ M, [SC4AD] = 5.0×10^−5^ M, [DBT] = 1.0×10^−6^ M and [NR] = 1.67×10^−6^ M).

# Reference

[1] H.-J. Wang; Y.-T. Li; M.-C. Shao; C.-S. Yu; J.-Y. Yang; L. Liu; S. Wang; J. Dou, Phototunable Phosphorescence Energy Transfer Based on Photodimerization of Coumarin-12-Crown-4 Accelerated by γ-Cyclodextrin. *Chem. Eng. J.* **2025**, *504*, 158772.

[2] X. Zhou; X. Zhao; X. Bai; Q. Cheng; Y. Liu, Thermal Activated Reversible Phosphorescence Behavior of Solid Supramolecule Mediated by β‐Cyclodextrin. *Adv. Funct. Mater.* **2024**, *34*, 2400898.

[3] F. Yang; M. Zhen; S. Wang; W. Wei; H. He; Y. Xu, Atropisomer-Based Construction of a New Perylene Diimide Macrocycle as Visible-Light Photocatalyst for Selective Sulfide Oxidation. *Chin. Chem. Lett.* **2022**, *33*, 5088-5091.
